# Investigation of hepatitis B virus mutations associated with immune escape and drug resistance in human immunodeficiency virus-infected patients

**DOI:** 10.12688/f1000research.132498.1

**Published:** 2023-09-27

**Authors:** Lorato Modise, Nomathamsanqa Sithebe, Hazel Mufhandu

**Affiliations:** 1 Biological Sciences, North West University, Mahikeng, North West, 2735, South Africa

**Keywords:** Hepatitis B virus, HIV, Drug resistance, Vaccine escape, Mutation, Co-infection

## Abstract

**Background:** HBV/HIV co-infection impact on high HBV replication, progression to liver cancer and high mortality. Co-infection may lead to cross-resistance of HBV and HIV drugs due to immune therapy pressure or hepatotoxicity. These challenges necessitate continuous monitoring of HBV variants to aid better diagnosis and treatment strategies. We conducted this study to characterise HBV among HIV infected individuals.

**Methods:** Serum was screened for HBsAg using ELISA, followed by DNA extraction, PCR amplification, Sanger sequencing and phylogenetic analysis.

**Results:** Of the 50 samples in this study 100% (N=50/50) were HBsAg positive; 78% (N=41/50) HBV/HIV co-infection and 92% (N=38/41) of the amplicons were successfully sequenced. Samples nucleotide sequences were identified as genotype A. Mutations prevalence in the HBsAg region was 47% (N=18/38); including mutations associated with diagnostic failure (K122R and T143S) and 7 vaccines escape mutations (P127T, G145R, S207N, Y200T, E164D, Y206H and L209V). Mutations showed resistance to lamivudine 71% (n=5/7), telbivudine 57% (n=4/7), 14% (n=1/7) for entecavir and 43% (n=3/7) for adefovir. Mutations causing resistance to lamivudine and telbivudine were M204V, L180M, V163I, and S202K; with S202K also causing resistance to entecavir and adefovir resistance mutation were I253Y, I223V and M250I. Multiple drug resistance mutations within a single sample contained L180M, M204V, S202K and M250I mutations. There was no statistical significance between the RT mutations associated with drug resistance at P>0.005. The correlation test exhibited a weak statistical association between SHB and RT mutations (0.877**).

**Conclusions:** This study shows the predominance of HBV genotype A in HIV-infected patients. We discovered HBV mutations linked to immune evasion and drug resistance. Although there is no statistical significance amongst the mutations associated with drug resistance and vaccine escape. These mutations could have clinical implications that could have therapeutic repercussions by influencing the correct clinical diagnosis and treatment in HBV/ HIV co-infected individuals.

## Introduction

The *Orthohepadnavirus* genus is a significant group of human viruses, and the Hepatitis B virus (HBV) is a prototype of the *Hepadnaviridae* family ( Summers *et al.*, 1975). Globally, it is thought that more than 300 million people are infected with HBV, which can lead to chronic liver disease and hepatocellular carcinoma (HCC), which accounts for over 1 million annual fatalities ( Musyoki *et al.*, 2015). The Baltimore virus taxonomy places HBV in the class VII of viruses ( Gust *et al*., 1986). It is an enclosed virus with 3200 nucleotide base pairs of partially antagonistic relaxed and circular double stranded DNA (RC-dsDNA), and it replicates through an intermediary ssRNA ( Liu *et al.*, 2021). Hepatocytes are the cells that the hepatitis B virus infects to cause liver infection, which can then clinically show as symptomatic or asymptomatic acute hepatitis marked by liver inflammation to fulminant hepatitis. Acute HBV hepatitis that is not treated may progress to chronic HBV hepatitis, which can cause cirrhosis and HCC ( Liang, 2009). Many tests, including clinical, biochemical, serological, and molecular approaches, are used to detect HBV infection, illness associated with HBV, and to differentiate between acute and chronic infections. The hepatitis B surface antigen (HBsAg), the hepatitis envelope antigen (HBeAg), the antibody to the hepatitis surface antigen (anti-HBs), the antibody to the hepatitis B core antigen (anti-HBc), and the immuglubullin antibody sub-class of the anti-HBc are the HBV antibodies and antigens that are determined by the serological testing (IgM anti-HBc). The key clinical marker for acute or chronic infection, prevalence, and endemicity of HBV infection is the presence of HBsAg, which can be identified two weeks after exposure. By identifying the qualitative and quantitative HBV-DNA, molecular methods can diagnose HBV. Based on the entire genome genetic variability of roughly 8%, HBV genomes have been divided into ten primary genotypes designating A to J ( Tatematsu *et al.*, 2009). The lack of a viral polymerase proofreading mechanism during viral replication causes the genetic heterogeneity ( Rahman *et al.*, 2016). Moreover, immunization and antiviral medication have an external and internal impact on HBV sequence heterogeneity. Geographical differences in the distribution of the various HBV genotypes, however, influence the pathogenicity of HBV and the administration of treatment. Due to their shared method of blood-born transmission, HBV, and the human immunodeficiency virus (HIV) frequently co-infect individuals ( Audsley *et al.*, 2010). Depending on where HBV is endemic, different geographic areas have different common pathways of transmission. Lamivudine (3TC) is used in the treatment of HBV and HIV ( Benson *et al.*, 2009). However, due to the development of drug resistance after 4 years of single-drug treatment, HIV-infected persons should not receive 3TC or emtricitabine (FTC) monotherapy for HBV infection ( Di Martino *et al.*, 1999). The emergence of the mutation rtm204 is a hallmark of 3TC resistance (YMDD mutation). The 3TC should only be administered to individuals on totally suppressive ART since it accelerates unchecked HBV DNA replication and raises the risk of death from HBV infection or illness. HBV prevalence has been documented in several studies among HIV-positive people from Durban, KwaZulu-Natal. However, investigations on the HBV genotype, HBV vaccine escape, and treatment resistance are still scarce in this field and most studies have concentrated on the seroprevalence. To ascertain the prevalence and genotypic characteristics of HBV among HIV-infected people in Durban, we undertook this study.

## Methods

### Study design, population, and ethical clearance

We employed the convenience sampling method and used samples that were available for use in this descriptive exploratory investigation; the sample size was not determined. Fifty preserved frozen sera samples from people who underwent HIV testing at the National Health Laboratory Services’ Inkosi Albert Luthuli Central Hospital (NHLS-IALCH-NHLS) in Durban, KwaZulu-Natal Province, South Africa, were utilized in this investigation. The 50 participants included both men and women who had HIV-positive test results. Participants were given a written informed consent form, the information on the consent form was given in a language that the patient understands which is English and Setswana. After receiving the written informed consent of all participants, samples were taken, tested for HIV positivity, and the residual sera were archived at the NHLS-IALCH-NHLS. At that moment, the subjects were not taking ART. The North-West University Research Ethics Regulation Committee issued an ethical certificate (Ref no: NWU-00068-15-A9) on 2015-05-22. Samples receival and data collection on the samples began in October 2015 to March 2016. The specimen’s codes were de-linked to keep patients anonymous; with only additional data on the age and gender of the study participants provided.

### Laboratory testing

**Samples collection and processing**

A total of 50 stored frozen serum specimens from HIV-infected individuals in Durban were donated from NHLS-IALCH-NHLS and travelled on ice to the virology laboratory at the North-West University. Upon arrival the specimens were aliquoted into numerous 1.5 mL Eppendorf tubes and stored at -80 °C until further use.

**Hepatitis B surface antigen (HBsAg) assay**

The Monolisa HBsAg ultra confirmatory kit was used in accordance with the manufacturer’s instructions to conduct an enzyme-linked immunosorbent test (ELISA) to identify the presence of HBV HBsAg (BioRad, Raymond Poincare, Marnes-la-Coquette, France). To identify and measure the presence of HBsAg, excess antibodies (anti-HBs; anti-HBs diluent: neutralization reagent) were used to neutralize 200 L of undiluted sera specimens. At 450 nm, the optical density (OD) index of the specimens was determined and compared to the COV mean of a negative control. Reactive specimens for HBsAg were defined as those with an index greater than or equal to the COV.

**DNA extraction of HBV**

Serum obtained from patients was used to extract HBV deoxyribonucleic acid (DNA) using the QIAamp DNA Mini kit (catalog number: 51304) from (Qiagen, Hilden, Germany) following the manufacturer’s instructions. This technique allows the isolation and purification of total DNA from contaminants, inhibitors, and nucleases in the serum. An aliquot of 200 μL of the serum sample was added into 1.5 μL Eppendorf tubes, to which 20 μL of proteinase K and 200 μL Buffer AL (binding buffer mixed with poly [A] carrier RNA) was added. An aliquot of 200 μL of the serum sample was added into 1.5 μL Eppendorf tubes, to which 20 μL of proteinase K and 200 μL of buffer AL (binding buffer mixed with poly (A) carrier RNA) were added. The mixture was pulse-vortex for 15 seconds to allow lysis of the mixture and destroy RNA, followed by a 10 minute incubation at 56 °C. The mixture was then transferred to a QIAamp spin column to allow binding of the DNA and centrifuged for 1 minute at 8 000 rpm. The column was placed into a clean collection tube, then 500 μL of buffer AW1 was added, and it was centrifuged for 1 minute at 8 000 rpm. The solution was aspirated, 500 μL of buffer AW2 was added to purify the DNA, and it was followed by centrifugation for 3 minutes at 14 000 rpm. The QIAamp spin column was placed in a sterile 1.5 μL Eppendorf tube, and 50 μL of elution buffer (provided by the kit as buffer AE) was added directly into the column and incubated at room temperature for 5 minutes to precipitate the DNA. The DNA was eluted by centrifugation at 8 000 rpm for 1 minute and stored at -20 °C until further analyses were performed. The negative control, consisting of nuclease-free water, was included in the extraction procedure to identify contamination.

### Polymerase chain reaction

**First round and nested-PCR**

A nested polymerase chain reaction (PCR) amplification of the overlapping surface/polymerase gene covering nucleotides 256 to 796 from *EcoRI* site was done as described previously ( Mphahlele, 2008) with slight modification. Outer sense strand forward primer, S1 (5′-CCT GCT GGT GGC TCC AGT TC-3′), and antisense strand reverse primer, S2Na (5′-CCA CAA TTC KTTGAC ATA CTT TCC A-3′) were used. The master mix were prepared using Ampli *Taq* Gold DNA polymerase (ThermoFisher Scientific, Waltham, Massachusetts, United States). For each sample the following reagent volumes and concentration of the master mix were prepared as follows: 18.5 μL nuclease free water, 2.5 μL of 1x PCR buffer with MgCl _2_, 0.5 μL (0.2 mM dNTP mix), 0.5 μL (10 μM) forward primer S1; 0.5 μL (10 μM) reverse S2Na anti-sense primer, 0.125 *Taq* DNA polymerase. A total of 22.5 μL of master mix was aliquoted into a 0.5 mL thin-walled PCR tube and 3 μL of DNA template was added. The PCR reaction mixtures (25.5 μL) was subjected to amplification of HBV DNA, carried out in an automated touch down thermal cycler CFX96 (Bio-Rad, Raymond Poincare, Marnes-la-Coquette, France). The HBV DNA amplification conditions were initial denaturation at 95 °C for 4 minutes, followed by 40× cycles involving denaturation at 95 °C for 4 minutes, annealing at 58 °C for 30 seconds, elongation at 72 °C for 1 minute, and final extension at 72 °C for 10 minutes.

**Nested PCR**

First round PCR product was used as a template for nested PCR. An aliquot of 3 μL of the first round PCR reaction was subjected to a nested PCR, the master mix volume and concentration were prepared as same for the first round PCR. The nested PCR conditions used were the same as first round PCR protocol except the annealing temperature at 55 °C for 45 seconds. Forward primers S6E (5′-GAGAAT TCCGAGGACTGG GGA CCC TG-3′) and reverse primer S7B (5′-CGG GAT CCT TAG GGT TTA AAT GTATAC C-3′) were used during nested PCR. The negative control consisting of nuclease free water and a positive control were included in the PCR amplification assays.

**PCR products verification**

PCR amplification products were verified using 1% agarose gel (ThermoFischer, Waltham, Massachusetts) stained with 0.15 U/μL ethidium bromide (Biorad, California, USA). Aliquot of 10 μL PCR amplicon product was mixed with 2 μL 10x loading buffer (ThermoFischer, Waltham, Massachusetts). The mixtures were run on 1% gel along with a 1 Kb Invitrogen molecular weight maker (ThermoFischer, Waltham, Massachusetts) as a band size reference. The agarose gel was run at 100 V for 45 minutes. The gel was placed inside the ultraviolet (UV) transilluminator (Bio-rad, Hercules, California, United States) to visualise and image capturing.

**Sequencing reaction**

The PCR products and the nested PCR primers S6E and S7B were sent for bi-directional Sanger sequencing at the Inqaba (Inqaba Biotechnological Industry, Pretoria, South Africa). The amplicons were prepared for direct sequencing using the BigDye Terminator v3.0 Cycle Sequencing Ready Reaction Kit (catalog number: 4458687) from (ThermoFischer, Waltham, Massachusetts). Briefly, an aliquot of 50 μL of the 1:1 ratio of sodium acetate: ethanol (NaAc:EtOH) was added to the amplicons solution and centrifuged at 2000 g for 30 minutes. The well plates were inverted and centrifuged at 150 g for 1 minute. Pre-chilled 70% ethanol was added into the wells, and then centrifuged at 2000 g for 5 minutes. The pellets were dried at 65 °C for 5 minutes and loaded into the sequencing machine, the ABI 3130XL genetic analyser (Applied Biosystems, Foster City, CA). An aliquot of 10 μL of the Hi-Di formamide was added into the sample for 5 minutes and loaded into a sequencing machine ABI 3130XL genetic analyser (Applied Biosystems, Foster City, CA).

### Sequences analysis

Nucleotide sequences of HBV chromatograms were viewed and edited by removing unwanted and mixed nucleotides character from the sequences by ChromasPro, version.1. The contiguous sequences were formed by joining overlapping DNA sequences of a gene using BioEdit. The consensus sequences were compared with complementary genotype sequences from the GenBank using the Basic local alignment search tool (BLAST). Representative sequences belonging to distinct genotypes were redeemed from GenBank to make comparisons with study sequences. Multiple sequence alignment of the sequenced nucleotide region was performed with ClustalW within the MEGA software package version 7.0 (TomHall, North Carolina State University). The aligned nucleotide base sequences were subjected to phylogeny inference on MEGA 7.0 ( Kumar *et al.*, 2004). The neighbour-joining method was used to generate dendograms, and the evolutionary relationship was performed using pairwise genetic distance with 1000 bootstrap replicates ( Saitou & Nei, 1987). Frequency estimates of evolutionary divergence between nucleotide sequences were then estimated using the Kimura 2-parameter model ( Kimura, 1980).

### Mutations analysis

The sequences were uploaded into the BioEdit and analysed for nucleotide base and amino acids changes. The sequences were further uploaded into the Geno2pheno to identify drug resistance mutations.

### Statistical analyses

Microsoft Excel and the data science statistical program STATA were used for data analyses (version 15). Excel was used to calculate the frequency of age as numeric values and the frequency of HBsAg and mutations as categorical and numeric variables. STATA was used to import the Excel (.csv) file to conduct additional research on the distribution and associations of the mutations. The likelihood of a link between the two categorical variables (mutations and age) was evaluated using the Fisher’s exact test. The Pearson correlation coefficient was used to examine the relationship between the surface region and the HBV virus’s reverse transcriptase mutations. In this study, a significant p value was defined as one with a 5% level of significance.

### Ethical approval

The study ethics certificate was provided by the North-West University research ethics regulatory committee (ref. NWU-00068-15-A9).

## Results

### Baseline demographic data

The baseline demographics of the study showed that all 50 HIV positive samples included were from patients of black African ethnicity most of the study participants were female at 66% (N = 33/50) and 34% (N = 17/50) were males as shown in ( Table 1). The median age and standard deviation of the study population were 33 years (range of 18-55).

**Table 1.** Demographics data of the patients by age, ethic group and ELISA screening of HBV and HIV antigens.

Age group by years	Ethnic group		HIV p24 antigen		HBsAg antigen	
	Female	Male	HIV + female	HIV + male	HBsAg + female	HBsAg + male
18-35	Black African	Black African	20	06	16	3
36-55	Black African	Black African	13	11	13	08
Total	33	17	33	17	29	11

### Laboratory testing

**HBsAg assay**

The HBsAg was positive in all HIV positive samples resulting in a 100% (N=50/50) HBV seroprevalence. Of the 50 HBsAg positive patients ( Table 1). Primarily, the study female’s participant had the highest HBsAg at (N=29/50) when compared to the males at (N=11/50) as shown in ( Table 1).

**PCR amplification**

**Figure 1.**
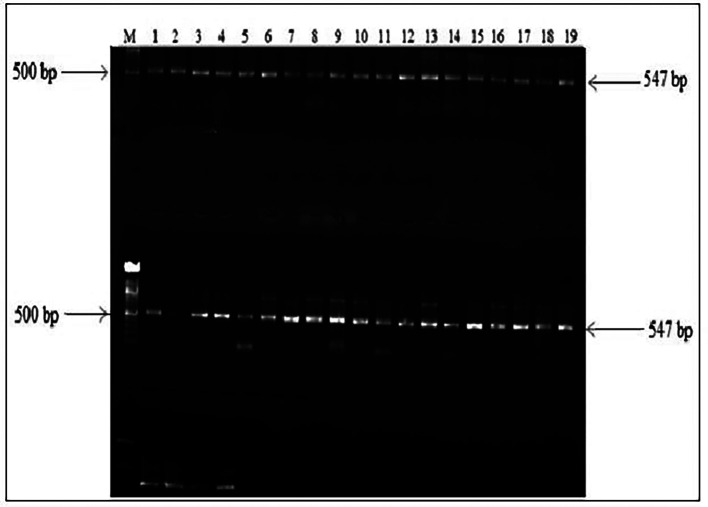
PCR amplification products of overlapping surface/polymerase HBV gene of this study. PCR amplicons are shown as 547 bp bands.

The PCR amplification of HBV DNA amplicons was successful in 78% (N=41/50) of the HBsAg and HIV positive samples. HBV overlapping surface/polymerase region amplicons are shown as 547 bp bands below ( Figure 1). PCR amplification could not be obtained for the other 22% (N=11/50) samples.

### Sequence analyses of overlapping surface/polymerase gene region

**Figure 2.**
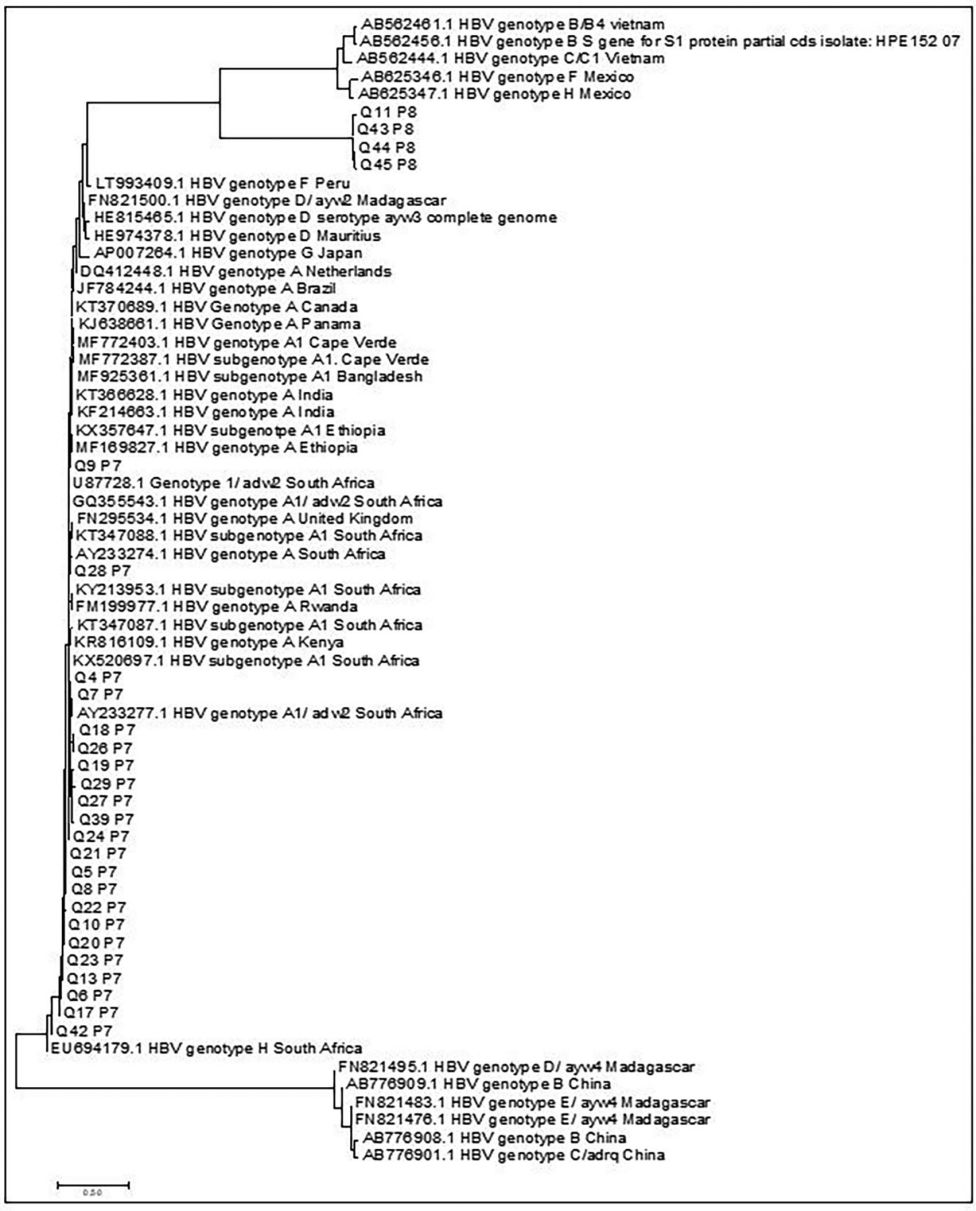
Phylogenetic tree comparing the overlapping surface/polymerase gene sequences of this study with representative sequences obtained from the GenBank (designated by accession numbers). Study sequences are represented by letters Q, P and followed by numeric values.

Only 92% (N=38/41) of the overlapping surface/polymerase nucleotide sequence products could be obtained by Sanger sequencing. The sample sequence showed a 98% to 99% homology similarity index with previously deposited HBV genotype A sequences in the GenBank. Phylogenetic tree analysis identified nucleotide sequences from this study as genotype A as depicted in ( Figure 2). The sub-genotypes of sequences were confirmed by depositing all the surface gene nucleotides sequenced into the Genotype2pheno database. The 38 individuals’ sequence were identified as sub-genotype A1 based on the results retrieved from the Geno2Pheno database with the percentage of similarity to sub-genotype profile of 96.85% -99.0%.

**Mutations within the surface gene**

The prevalence of mutations in the surface gene was 47% (N=18/38) and mutations were found in the “α” epitope, “β”-cell epitope, “T” helper cell epitope and outside the “α” epitope as shown in ( Table 2). The most common mutations on the surface region of HBV were S207N at 71% (27/38), followed by L216V and A194V at 23%, P70H at 21% L209V at 18%, P217L at 8%, F134L, E164D and T189I at 5% and S204R, S117N, T143S, G145R, Y206H, P127T, Y200T, F129T and K122R all at 3% ( Table 2).

**Table 2.** Mutations within the surface gene region.

Epitope	Mutation	Frequency (%)	Function
“α” epitope	K122R	3	Sub-serotype change (d/y)
	F134L	5	Unclear
	S117N	3	Unclear
	T143S	3	Vaccine escape selection
“β”-cell epitope	S207N	71	Unknown in genotype A
	Y200T	3	Unknown in genotype A
	G145A	3	Vaccine escape selection
T-helper epitope	P127T	3	Lower reactivity in HBsAg assay
Outside “α”	E164D	5	Vaccine-escape mutation
	L209V	18	
	Y206H	3	
	L216V	23	
	A194V	23	
	P70H	21	Overt HBV infections
	P127L	8	
	T189I	5	
	S204R	3	
	F129T	3	

**Mutations within the polymerase gene region**

Mutations prevalence were reported at 36% (N=14/38) in different positions within the reverse transcriptase (RT) region. The M129L mutation was the most common in this study, accounting for 84% (32/38), followed by V163I at 78%; I253Y at 50% and S105T at 40%. Other mutations identified from sequences included L217R, A233S, Q125E, T128A, V214A, V204I, M204V, L180M, V173L and S202K at 21%, 16%, 13%, 8% and 3% ( Table 3). The prevalence of mutations associated with drug resistance was 57% (8/14) within the RT region ( Table 3). Drug resistance mutations included lamivudine (LMV) resistance at 71% (5/7), telbivudine (LdT) at 57% (4/7), 14% for entecavir (ETV) and 43% for adefovir (ADV) resistance. Mutations causing resistance to LMV and LdT were M204V, L180M, V163I, and S202K. S202K mutation also causes resistance to ETV. ADV resistance mutations were I253Y, I223V and M250I ( Table 3). Multiple drug resistance mutations within a single sample were identified from one sample containing L180M, M204V, S202K and M250I mutations.

**Table 3.** Distribution of amino acids substitution in reverse transcriptase region of polymerase of HBV positive patients co-infected with HIV.

Amino acids substitutions	Frequency
**Drug resistance mutation**	
M129L	84%
M204V	3%
L180M	3%
V163I	78%
V173L	3%
A223S	16%
Q125E	13%
S202K	3%
L217R	21%
V214A	3%
V204I	3%
I253Y	50%
T128A	8%
S105T	40%

**Frequency of mutations**

The prevalence of the mutations on the sequences of surface protein region was (N=39) and RT mutations were (N=41). The mean and standard deviation of the SHB mutations was 7.66±4.79; for RT mutations the mean and standard deviation was 11.21±5.44 as depicted in ( Table 4).

**Table 4.** Frequencies of the resistance of mutations in the polymerase of HBV samples.

Drug	Resistance		Susceptible		Resistance	Susceptible	Probability
	Age 18 – 35	Age 36-55	Age 18-35	Age 36-55	Total	Total	Fishers extract test
Lamivudine	3 12.00	3 17.65	22 88.00	14 82.35	6	36	0.6720
Adefovir	1 4.00	0 0.00	24 96.00	17 100.00	1	42	1.0000
Entecavir	3 12.00	3 17.65	22 88.00	14 82.35	6	36	0.6720
Tenofovir	0	0	25 100.00	17 100.00	0	42	0.6355

**Association of mutations on the surface and polymerase**

**Figure 3.**
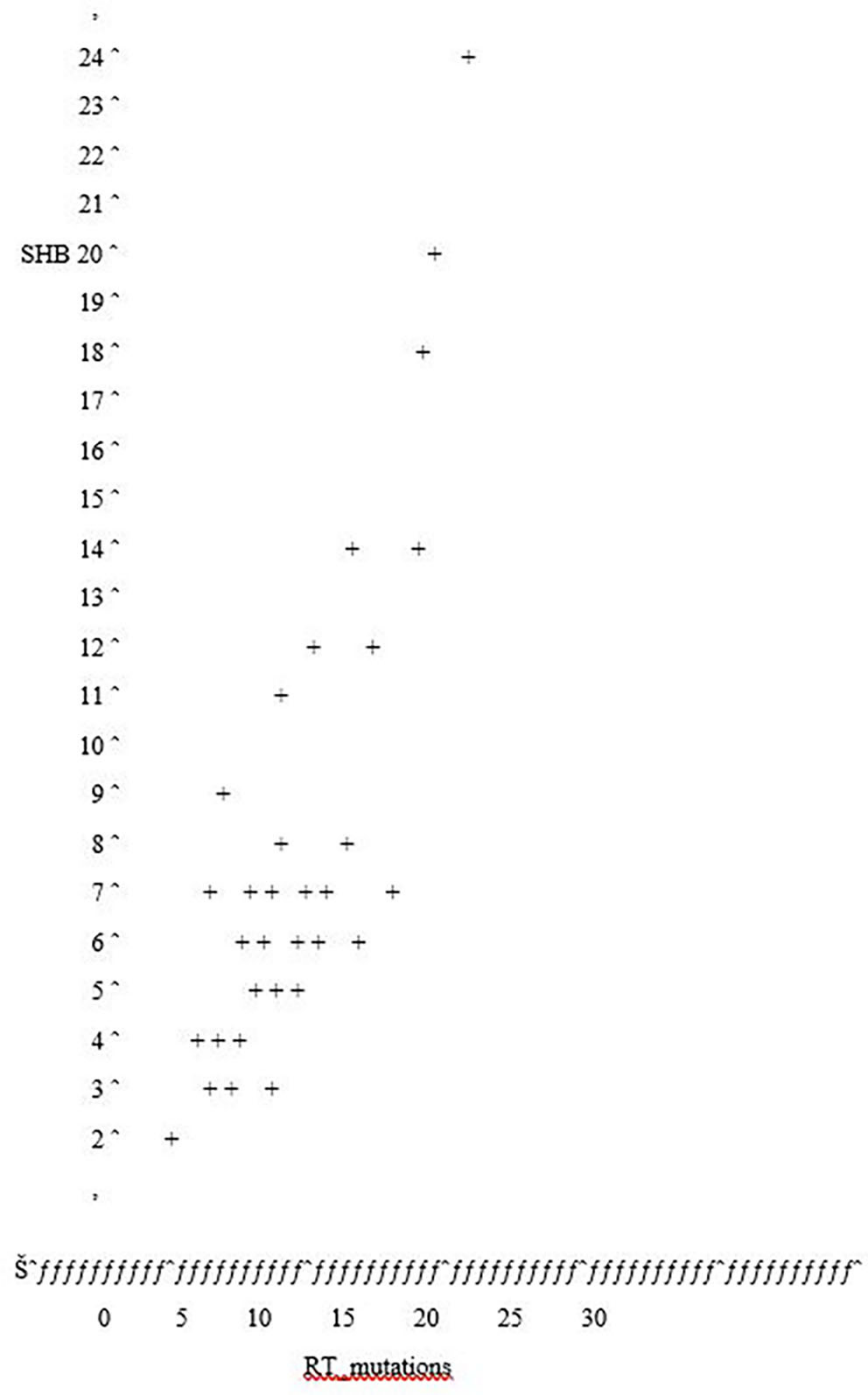
Scatter plot showing the correlation between the SHB and RT mutations. Plot of SHB_protein *RT_mutations, symbol used is ‘+’. 11 obs had missing values. 7 obs hidden.

A correlation test was done between the SHB and RT mutations was done using and the Pearson Correlation. There was no statistical significance between the RT mutations at P>0.005 ( Table 4). The correlation test exhibited a weak statistical association between SHB and RT mutations (0.877**) as shown in Figure 3.

## Discussion

In the current study, the HBsAg seroprevalence in HIV-infected patients was 100%. The HBsAg prevalence in HIV-infected people is higher in this study as compared to previous studies in South Africa, with an estimation between 3% and 23% reported from the Western Cape, KwaZulu-Natal, and Gauteng provinces ( Andersson *et al.*, 2013; King & Hagemeister, 2016; Lukhwareni *et al.*, 2009; Maponga, 2012; Mphahlele, 2008; Msomi *et al.*, 2020; Samsunder *et al.*, 2019). The HBsAg seroprevalence from this study is not coherent with published data from South Africa; comparing with previous studies, they used larger sample cohort in their studies, whereas our study used a small sample size, which influenced the high prevalence rate. However, the study report supports previous studies that endemic HBV is still present in South Africa and that HBV seroprevalence among HIV-infected people is presently high among adults ( Amponsah-Dacosta, 2021; Maepa *et al.*, 2022). All the HBsAg-positive samples were subjected to PCR amplification of the HBV DNA. The partially overlapping surface/polymerase gene region was successfully amplified in 78% (41/50), confirming the active replication of HBV and HBV/HIV co-infection on the input study samples. The presence of HBV/HIV co-infection is attributed to the shared route of viral transmission since both viruses share a similar route of transmission. Furthermore, it was also reported that females were the majority infected with HBV infection at 66% (N=33/50) compared to their male counterparts at 34% (N=17/50) in this population. This does not correlate with the findings of ( Kew *et al.*, 2008), who reported a prevalence of 4% in women compared with 9% in men. Similarly ( Moonsamy *et al.*, 2022), reported higher prevalence rates in males than females aged 25 to 49 years. The phylogenetic analysis shows the predominance of genotype A from our study sequences. The identification of HBV genotype A is coherent with previous studies in South Africa ( Kimbi *et al.*, 2004; Kramvis, 2014). The patient’s sequences were further identified as sub-genotype A1 by Geno2Pheno, indicating that HBV showed significant diversity, suggesting an African origin. The HBsAg serves is part of the S region which is employed in the development of recombinant vaccines; this region consists of α, β and T–cell epitopes. The “α” epitope is the primary area that neutralizing antibodies attacks and it contains the β and T–cell epitopes ( Caligiuri *et al.*, 2016). The “a” determinant, also known as the primary hydrophilic region of HBsAg, is the collection of B cell epitopes and is made up of the amino acids 124–147 ( Liu *et al.*, 2017). The “α” epitope domain of HBsAg may undergo mutations due to nucleotide and amino acid substitutions, which may result in structural and functional alterations in the S protein ( Zuckerman & Zuckerman, 2003). This could alter the antigenicity of the vaccination and result in mutations that escape the immunization, allowing mutant HBV to replicate in the body of the vaccinated population ( Yan *et al.*, 2017). We determined the mutations on the partial surface gene of HBV/HIV co-infected individuals from our study. A total of 18 nucleotide substitutions were observed within the HBsAg region in all 38 partial surface sequences. The study reports mutations at sequence locations at the at α, β and T–cell epitopes that have clinical ramifications. The primary hydrophilic region (aa79 to aa147) of the surface antigen had the “α” epitope and contained four mutations (K122R, F134L, T143S, and S117N). G145R, S207N, and Y200T variations were found in the "T" epitope, whereas P127T was found in the “β” epitope of the “α” determinant region. The following mutations were found to be absent from the “α” epitope: E164D, L209V, Y206H, A194V, P70H, P127L, T189I, S204R, and F129T. We found escape mutations from vaccines including P127T, G145R, S207N, Y200T, Y206H, and L209V. Among 27 samples, the S207N mutation was the most prevalent one. P127T has been linked to vaccination escape selection in the past ( Colagrossi *et al.*, 2018); which contradicts the findings of Kuzin *et al.*, (2012), who reported this mutation to causing diagnostic failure. The clinical implications of this mutations must further be explored *in vitro.* One patient sequence contained the mutation G145R, which has already been identified by ( Colson *et al.*, 2007; Yan *et al.*, 2017). In the “β”-cell epitope of HBsAg, the G145R mutation is a significant vaccine escape mutation that is linked to a point mutation that results in the substitution of arginine for glycine at position 145 (G145R). The HBsAg antigenicity is decreased because of the interference with antibody binding ( Kao & Chen, 2002; Su *et al.*, 2008). Given that it was the first vaccine-escape mutation discovered in children who received vaccinations in Italy, the G145R mutation is a typical mutation ( Zanetti *et al.*, 1988). Its occurrence and stability have increased over the years with an increase of HBV endemicity and use of universal immunization ( Caligiuri *et al.*, 2016). This mutation is crucial in causing failure on the detection of HBV by serological routine assays ( Cooreman *et al.*, 2001). The long-term success of mass vaccination, however, has been previously reported to be threatened by G145R, which has grown more dominant with vaccine escape selection ( Yan *et al.*, 2017 ). Thus, it is advised that it be incorporated into the design of future vaccines ( Yan *et al.*, 2017). It is said that this mutation has a possible horizontal transmission pattern ( Colagrossi *et al.*, 2018). Globally, the G145R mutations have previously been linked to HBV breakthrough infections in the population who had received vaccinations ( Cooreman *et al.*, 2001; Theamboonlers *et al.*, 2001). On the other hand, mutations G145R and F143S have also been discovered in people with occult hepatitis B infection (OBI), and they have been associated to the clinical implications. On the other hand, mutations G145R and F143S have also been found in individuals with occult hepatitis B infection (OBI), and they have been linked to the clinical implications of diagnostic failure due to suboptimal HBsAg detection when using commercial ELISA kits ( Coppola *et al.*, 2016). It was previously reported as a nucleos (tide) analog (NA-induced) immune escape mutation ( Colagrossi *et al.*, 2018; Lai *et al.*, 2003), demonstrated that the E164D result from the substitution change in the overlapping polymerase region caused by the mutation rtM204V, rtM204I, and rtV173L) ( Lai *et al.*, 2003; Zöllner *et al.*, 2001). E164D was another significant mutation discovered from this study. These mutations produce a decrease in anti-HBs and are linked to lamivudine resistance therapy ( Colagrossi *et al.*, 2018). Additional mutations found in this study included K122R, T143S, Y206H, L209V, S207N, Y200T, and P70H. Nevertheless, there is little information available about these mutations’ effects on surface protein structure and activities, and more research is needed to determine how they may affect patients’ health. K122R and T143S mutations, on the other hand, have reportedly been linked to diagnostic adequacy ( Cooreman *et al.*, 2001). With certain ELISA diagnostic methods failing to identify HBsAg from samples bearing the K122R in prior research ( Malik *et al.*, 2012). All the HBsAg mutations mentioned above have clinical implications that are connected to immunological escape and have an impact on the antibodies’ ability to recognize HBsAg. whilst others are connected to diagnostic failure. Antiviral medications that prevent reverse transcription specifically target the HBV polymerase to prevent HBV DNA replication. The nucleoside/nucleotide analogues LMV, ADV, TDF, LdT, and ETV are antiviral medications that are used to treat HBV polymerase. Despite the introduction of TDF, lamivudine treatment is still favoured among patients with HBV/HIV co-infection in South Africa. Antiviral medications may potentially contribute to the development of drug resistance due to selection pressure during long-term use of the antiviral treatment, in addition to the high mutation rate caused by the HBV error-prone ( Fares & Holmes, 2002). The frequency of drug resistance-related mutations in the RT region was 50% (N=7/14). M204V, L180M, V163I, and S202K were mutations linked to LdT resistance; S202K also conferred ETF resistance. Combining L180M and M204V results in increased cross-resistance to other nucleosides and decreased sensitivity to ETV but not ADV. It may also result in vaccine escape mutations in the overlapping S-region, which would stop HBsAg from secreting ( Sheldon *et al.*, 2005). ADV resistance mutations included the I253Y, I223V, and M250I mutations. A single sample from one patient that contained the mutations L180M, M204V, S202K, and M250I was shown to have several drug resistance mutations. All the drug resistance mutations found in the RT of the pol region were found in genotype A sequences. Moreover, the following compensatory mutations were found: S202K, Q125E, L217R, V124A, V204I, I253Y, T128A, and S105T. We still need to investigate how these changes affect RT functions. As this study found no mutations linked to tenofovir resistance, we advise using a tenofovir-based regimen to treat HBV in people who also have HIV. At P>0.05, there was no statistical difference between the mutations linked to medication resistance. The Pearson Correlation was used to conduct a correlation test between the SHB and RT mutations. The connection between SHB and RT mutations was not strong. Further *in vitro* studies must be carried out to investigate the effects of the detected alterations on the antibody neutralization and nucleoside/tide drug analogues. This does not rule out the possibility that these mutations have major clinical implications towards the diagnosis and therapy.

## Conclusion

This study demonstrates the prevalence of HBV genotype A in HIV-positive patients as well as the presence of HBV mutations in HBV/HIV co-infected people. We discovered HBV mutations linked to immune escape and drug resistance that could have therapeutic repercussions by influencing the correct clinical diagnosis and HBV treatment. This study has improved our understanding of the genotypes and mutations of HBV that have clinical implications in those who have HBV and HIV co-infection in South Africa. To determine how immune escape and drug resistance-related HBV mutations affect diagnosis and drug resistance treatment in the context of HIV/HVB co-infection, we recommend testing the HIV samples for these mutations.

## Data Availability

Figshare: data set for patients’ demographics, HIV and HBV test 2017-2019.xlsx, https://doi.org/10.6084/m9.figshare.23946621.v1 ( Modise, 2023a). This project contains the following underlying data:
- data set for HIV 2017-2019.xlsx - data set for demographic and virology test 2017-2019.xlsx Figshare: PCR amplicon gel electrophoresis, https://doi.org/10.6084/m9.figshare.23815278.v1 ( Modise, 2023b). Figshare: HBV PCR amplicon gel image.pdf, https://doi.org/10.6084/m9.figshare.23946639.v1 ( Modise, 2023c). Figshare: Reference sequences accession number and Genotype, https://doi.org/10.6084/m9.figshare.23946642.v1 ( Modise, 2023d). Data are available under the terms of the Creative Commons Attribution 4.0 International license (CC-BY 4.0).
