# Investigation of hepatitis B virus mutations associated with immune escape and drug resistance in human immunodeficiency virus-infected patients

**DOI:** 10.12688/f1000research.132498.2

**Published:** 2024-05-30

**Authors:** Lorato Modise, Nomathamsanqa Sithebe, Hazel Mufhandu

**Affiliations:** 1 Biological Sciences, North West University, Mahikeng, North West, 2735, South Africa

**Keywords:** Hepatitis B virus, HIV, Drug resistance, Vaccine escape, Mutation, Co-infection

## Abstract

**Background:**

Co-infection of hepatitis B virus (HBV) and human immunodeficiency virus (HIV) has an impact on high HBV replication and progression to liver cancer. These may lead to cross-resistance of drugs due to therapeutic pressure or liver toxicity. These require continuous monitoring of HBV variants for better diagnosis and treatment strategies.

**Methods:**

Convenience sampling was used to collect fifty archival sera from Inkosi Albert Luthuli Central Hospital. The Sera were subjected to HBsAg screening using ELISA, DNA extraction, PCR amplification, Sanger sequencing, phylogenetic and mutation analysis. A correlation test was performed to measure the association between polymerase and surface mutations.

**Results:**

Of the 50 samples, 86% (N= 43/50) were HBsAg positive; 82% (N=41/50) PCR positive and 92% (N=38/41) sequenced. The HBV sequences showed similarity to genotype A (73% [N=19/26]) and (24% [N=7/26]) as genotypes B, C, D, E, F, and G. Prevalence of the mutations in the Surface region was (47% [N=18/38]); including diagnostic failure (K122R and T143S) and vaccines escape mutations (P127T, G145R, S207N, Y200T, E164D, Y206H and L209V). The mutations in the RT was at (36% [N=14/38]) with drug resistance mutations (DRM) at (50% [7/14]). Mutations showed resistance to lamivudine (LMV) at (35% [5/14]), telbivudine (LdT) at (29% [4/14]), (14% [2/14]) for entecavir (ETV) and (21% [3/14]) for adefovir (ADV). One sample had a combination of L180M, M204V, S202K, and M250I mutations. There was no statistical significance between S and RT mutations at P>0.005 and the statistical correlation between RT and SHB mutations was weak at 0.877.

**Conclusions:**

Our findings highlight the prevalence of HBV genotype A in HIV-infected patients in South Africa. We provide evidence of mutations linked to immune evasion and drug resistance. Mutations have no statistical significance but can have clinical Implication on the diagnosis and treatment of HBV in HBV/HIV co-infected individuals.

## Introduction

The *Orthohepadnavirus* genus is a significant group of human viruses, and the HBV is a prototype of the *Hepadnaviridae* family ( Summers *et al.*, 1975). More than 300 million people worldwide are chronically infected with hepatitis B, which can lead to severe liver disease and hepatocellular carcinoma (HCC), which accounts for more than 1 million annual deaths ( Musyoki *et al.*, 2015). HBV/HIV co-infections in the region of South Africa (SA) are estimated to range from 5% to 23% ( Anastasiou *et al*., 2017; Kaswa & de Villiers, 2023; King & Hagemeister, 2016). However, only limited evidence is available for co-infection in KwaZulu-Natal (KZN) province with a hepatitis B surface antigen (HBsAg) seroprevalence of 8.5% reported in HIV-infected people ( Msomi *et al*., 2020). The introduction of the hepatitis B vaccine into the South African Expanded Program of Immunization (EPI) in 1995 led to a reduction in liver diseases ( Spearman *et al*., 2013). Treatment for co-infection with HBV/HIV consists of a combination of tenofovir/(TDF) and lamivudine (LMV)/emtricitabine (FTC) /efavirenz (EFV) according to ( Maponga *et al*., 2020). However, treatment may be threatened by the appearance of mutations that could cause unfavorable clinical effects, such as vaccine escape and drug resistance. There is also little information on the molecular characteristics and mutations of HBV in the HIV-infected people in SA. Several studies have found M204V/I in the YMDD motif (amino acid 203±206) among HIV-infected drug-naive and drug-experienced people ( Mokaya *et al*., 2018; Selabe *et al*., 2007; Selabe *et al*., 2009). Early studies reported drug resistance resulting from a single or combination of the following mutations (V173L, L180M, M204V/I, and A181V/T to lamivudine), (rtA181V/T and rtN236T to adefovir) or (rtI169T, rtI184G, rtS202G/I and rtM250V to entecavir) by ( Colagrossi *et al*., 2018; Lai *et al*., 2003; Torresi, 2002; Zöllner *et al*., 2001). DRM in polymerase (Pol) region of HBV can lead to the emergence of immune escape mutations in the major hydrophilic region (MHR) and vice versa. The surface region (S) serves as a part that is used in the development of recombinant vaccines and for the routine serological marker diagnosis of HBV. This region consists of the MHR and incorporates the ‘a’ determinant region within amino acid: aa124-aa147 ( Liu *et al*., 2017). Previous studies have reported mutations at this location and described them as having clinical implications such as vaccine escape and diagnostic failure ( Adesina *et al*., 2021; Cooreman *et al*., 2001). The prevalence of HBV has been documented in several studies among HIV positive people in KZN ( Millar *et al*., 2023; Samsunder *et al*., 2019). However, investigations on the HBV genotype, mutations associated with vaccine escape and drug resistance are still scarce in this region, and most studies have focused on reporting seroprevalence. The aim of the study was to describe the prevalence and molecular characterization of HBV mutations associated with immune escape and drug resistance in HIV-infected individuals in Durban, KZN, South Africa.

## Methods

### Study design and population

We applied a convenience sampling method to collect fifty archival sera in this descriptive exploratory investigation. The sample size was not determined, and we used samples that were already available. Sera were from people who underwent HIV testing at the Inkosi Albert Luthuli Central Hospital (NHLS-IALCH-NHLS) in Durban, KwaZulu-Natal Province, South Africa. Based on the sample size of 50 sera, we used the confidence level of 95% and the margin of error for our study was 14%. The 50 participants included men and women who previously tested positive for HBsAg and HIV. Participants received a written informed consent form, the information on the consent form was given in a language the patient understands, which is English and their native language (Zulu). Demographic data (age, sex, and ethnicity) were also collected. The specimen’s codes were de-linked to keep patients anonymous; with only additional data on the age and gender of the study participants provided.

### Laboratory testing

***Hepatitis B surface antigen (HBsAg) assay***

The Monolisa HBsAg ultra confirmatory kit was used in according to the manufacturer’s instructions to perform an enzyme-linked immunosorbent assay (ELISA) on samples previously positive for HBsAg to confirm the presence of HBsAg marker (BioRad, Raymond Poincare, Marnes-la-Coquette, France). To identify and measure the presence of HBsAg, excess antibodies (anti-HBs; anti-HBs diluent: neutralization reagent) were used to neutralize 200 μL of undiluted sera specimens. At 450 nm, the optical density (OD) index of the sample was determined and compared to the COV mean of a negative control. Reactive specimens for HBsAg were defined as those with an index greater than or equal to the COV.

***DNA extraction of HBV***

The sera obtained from patients were used to extract HBV deoxyribonucleic acid (DNA) using the QIAamp DNA Mini kit (catalog number: 51304) from (Qiagen, Hilden, Germany) following the manufacturer’s instructions. This technique allows the isolation and purification of total DNA from contaminants, inhibitors, and nucleases in the serum. An aliquot of 200 μL of the serum was added into 1.5 μL Eppendorf tube, to which 20 μL of proteinase K and 200 μL Buffer AL (binding buffer mixed with poly [A] carrier RNA) was added. The mixture was pulse-vortex for 15 seconds to allow lysis of the mixture and destroy RNA, followed by a 10 minute incubation at 56 °C. The mixture was then transferred to a QIAamp spin column to allow binding of the DNA and centrifuged for 1 minute at 8 000 rpm. The column was placed into a clean collection tube, then 500 μL of buffer AW1 was added, and it was centrifuged for 1 minute at 8 000 rpm. The solution was aspirated, 500 μL of buffer AW2 was added to purify the DNA, and it was followed by centrifugation for 3 minutes at 14 000 rpm. The QIAamp spin column was placed in a sterile 1.5 μL Eppendorf tube, and 50 μL of elution buffer (provided by the kit as buffer AE) was added directly into the column and incubated at room temperature for 5 minutes to precipitate the DNA. The DNA was eluted by centrifugation at 8 000 rpm for 1 minute and stored at -20 °C until further analyses were performed. The negative control, consisting of nuclease-free water, was included in the extraction procedure to identify contamination.

### Polymerase chain reaction

***First round and nested-PCR***

A nested polymerase chain reaction (PCR) amplification of the overlapping surface/polymerase gene covering nucleotides 256 to 796 from *EcoRI* site was done as described previously ( Mphahlele, 2008) with slight modification. Outer sense strand (forward primer) S1 (5′-CCT GCT GGT GGC TCC AGT TC-3′), and anti-sense strand (reverse primer) S2Na (5′-CCA CAA TTC KTTGAC ATA CTT TCC A-3′) were used. The master mix were prepared using Ampli *Taq* Gold DNA polymerase (ThermoFisher Scientific, Waltham, Massachusetts, United States). For each sample the following reagent volumes and concentration of the master mix were prepared as follows: 18.5 μL nuclease-free water, 2.5 μL of 1x PCR buffer with MgCl _2_, 0.5 μL (0.2 mM dNTP mix), 0.5 μL (10 μM) forward primer S1; 0.5 μL (10 μM) reverse S2Na anti-sense primer, 0.125 *Taq* DNA polymerase. A total of 22.1 μL of master mix was aliquoted into a 0.5 mL thin-walled PCR tube and 3 μL of DNA template was added. The PCR reaction mixtures (25.1 μL) was subjected to amplification of HBV DNA, carried out in an automated touch down thermal cycler CFX96 (Bio-Rad, Raymond Poincare, Marnes-la-Coquette, France). The HBV DNA amplification conditions were initial denaturation at 95 °C for 4 minutes, followed by 40× cycles involving denaturation at 95 °C for 4 minutes, annealing at 58 °C for 30 seconds, elongation at 72 °C for 1 minute, and final extension at 72 °C for 10 minutes.

***Nested PCR***

First round PCR product was used as a template for nested PCR. An aliquot of 3 μL of the first round PCR reaction was subjected to a nested PCR, the master mix volume and concentration were prepared as same for the first round PCR. The nested PCR conditions used were the same as first round PCR protocol except the annealing temperature at 55 °C for 45 seconds. Forward primers S6E (5′-GAGAAT TCCGAGGACTGG GGA CCC TG-3′) and reverse primer S7B (5′-CGG GAT CCT TAG GGT TTA AAT GTATAC C-3′) were used during nested PCR. The negative control consisting of nuclease-free water and a positive control were included in the PCR amplification assays.

***PCR products verification***

PCR amplification products were verified using 1% agarose gel (ThermoFischer, Waltham, Massachusetts) stained with 0.15 U/μL ethidium bromide (Biorad, California, USA). Aliquot of 10 μL PCR amplicon product was mixed with 2 μL 10x loading buffer (ThermoFischer, Waltham, Massachusetts). The mixtures were run on 1% gel along with a 1 Kb Invitrogen molecular weight maker (ThermoFischer, Waltham, Massachusetts) as a band size reference. The agarose gel was run at 100 V for 45 minutes. The gel was placed inside the ultraviolet (UV) transilluminator (Bio-rad, Hercules, California, United States) to visualise and image capturing.

***Sequencing reaction***

The PCR products and the nested PCR primers S6E and S7B were sent for bi-directional Sanger sequencing at the Inqaba (Inqaba Biotechnological Industry, Pretoria, South Africa). The amplicons were prepared for direct sequencing using the BigDye terminator v3.0 cycle sequencing ready reaction kit (catalog number: 4458687) from (ThermoFischer, Waltham, Massachusetts). Briefly, an aliquot of 50 μL of the 1:1 ratio of sodium acetate: ethanol (NaAc:EtOH) was added to the amplicons solution and centrifuged at 2000 g for 30 minutes. The well plates were inverted and centrifuged at 150 g for 1 minute. Pre-chilled 70% ethanol was added into the wells, and then centrifuged at 2000 g for 5 minutes. The pellets were dried at 65 °C for 5 minutes followed by an addition of 10 μL Hi-Di formamide for 5 minutes and loaded into the sequencing machine ABI 3130XL genetic analyser (Applied Biosystems, Foster City, CA).

### Sequences analysis

Nucleotide sequences of HBV chromatograms were viewed and edited by removing unwanted and mixed nucleotides character from the sequences by ChromasPro, version.1. The contiguous sequences were formed by joining overlapping DNA sequences of a gene using BioEdit. The consensus sequences were compared with the GenBank complementary genotype sequences using the Basic local alignment search tool (BLAST). Representative sequences belonging to different genotypes were redeemed from GenBank to make comparisons with the study sequences. Multiple sequences alignment was performed with ClustalW within the MEGA software package version 7.0 (TomHall, North Carolina State University). The aligned nucleotide base sequences were subjected to phylogeny inference on MEGA 7.0 ( Kumar *et al.*, 2004). The neighbour-joining method was used to generate a phylogenetic dendrogram, to describe the identity of the virus and the evolutionary relationship ( Saitou & Nei, 1987). Frequency estimates of evolutionary divergence between nucleotide sequences were then estimated using the Kimura 2-parameter model ( Kimura, 1980).

### Mutations analysis

The aligned sequences were uploaded into the BioEdit and analysed for nucleotide base and amino acids changes. The sequences were further uploaded into the Geno2pheno to identify DRMs in the reverse transcriptase (RT) of polymerase and mutations in the overlapping S region.

### Statistical analyses

Microsoft Excel and the data science statistical program STATA were used for data analyses (version 15). Excel was used to calculate the frequency of age as numeric values and the frequency of HBsAg and mutations as categorical and numeric variables. STATA was used to import the Excel (.csv) file to conduct additional analysis on the distribution and association of the mutations. The likelihood of a link between the two categorical variables (mutations and HBsAg) was evaluated using the Fisher’s exact test. The Pearson correlation coefficient was used to examine the relationship between the surface region and RT mutations of HBV. In this study, a significant p value was defined as one with a 5% level of significance.

### Ethical approval

The study ethics certificate was provided by the North-West University research ethics regulatory committee (ref. NWU-00068-15-A9).

## Results

### Baseline demographic data

The baseline demographics of the study showed that all 50 HIV positive samples included were from patients of black African ethnicity most of the study participants were female at 64% (N=32/50) and 36% (N=18/50) were males as shown in Table 1. The median age and standard deviation of the study population were 33 years (range of 18-55).

### Laboratory testing

***HBsAg assay***

The confirmatory screening of HBsAg reported a prevalence of 86% (N=43/50) with 12% (N=6/50) being negative and 2% (N=1/50) missing data ( Table 1). Majority of the female’s participant were HBsAg positive at 58% (N=29/50) when compared to the males at 28% (N=14/50) as shown in Table 1.

**Table 1.** Demographics data of the patients by age, ethic group, HBV and HIV antigens markers.

Age group by years	Ethnic group		HIV p24 antigen		HBsAg antigen	
	Female	Male	HIV + female	HIV + male	HBsAg + female	HBsAg + male
18-35	Black African	Black African	20	07	17	06
36-55	Black African	Black African	12	11	12	08
Total	32	18	32	18	29	14

***PCR amplification***

The PCR amplification of HBV DNA amplicons was successful in 82% (N=41/50) of the samples. HBV overlapping surface/polymerase region amplicons. PCR amplification could not be obtained for the other 18% (N=09/50) samples.

***Sequence analyses of overlapping surface/polymerase regions***

**Figure 1.**
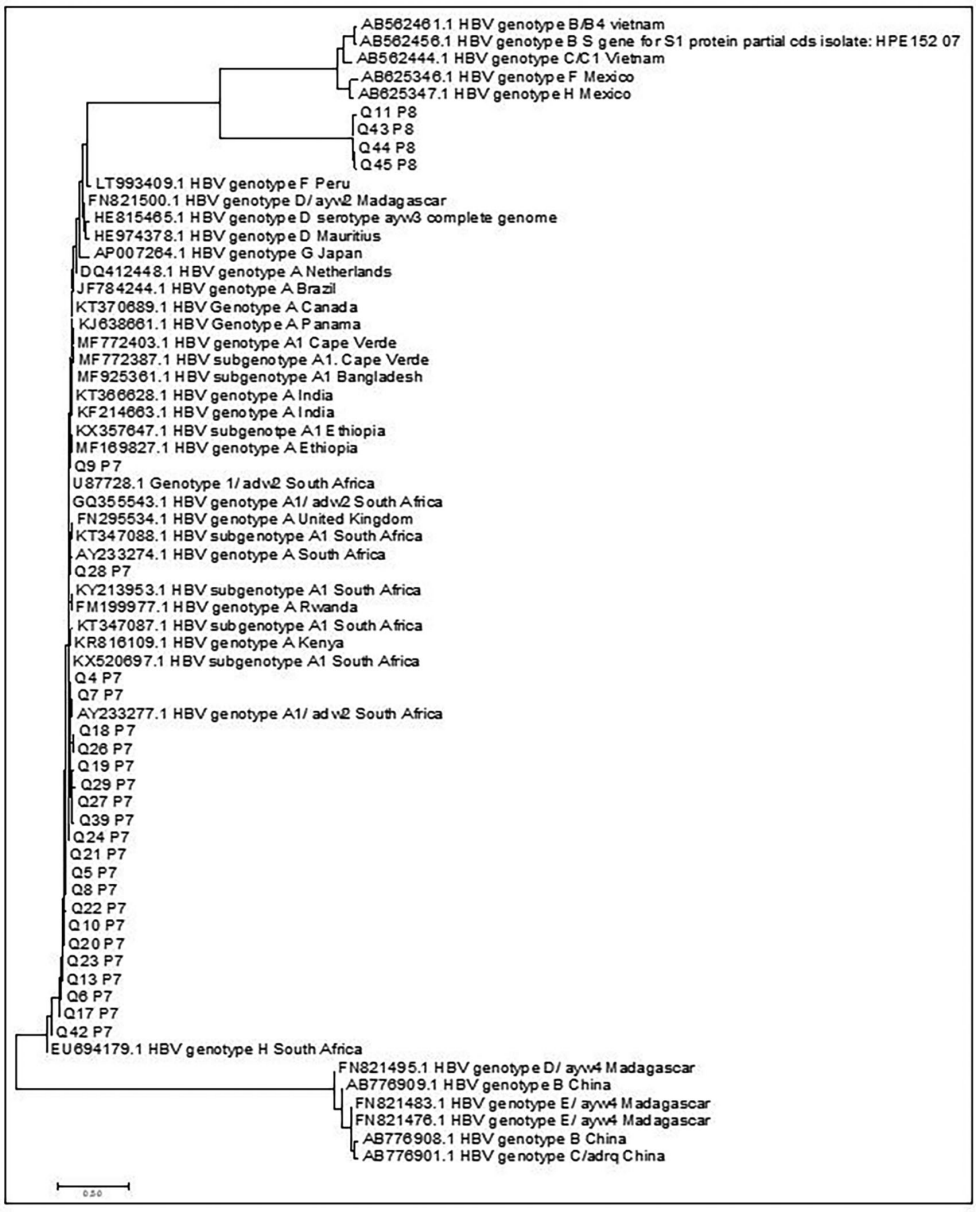
Phylogenetic tree comparing the overlapping surface/polymerase gene sequences of this study with representative sequences obtained from GenBank (designated by accession numbers). The study sequences are represented by the letters Q, P and are followed by numeric values.

Only 92% (N=38/41) sequence products could be obtained by Sanger sequencing. Only 26 sequences were used to perform phylogeny analysis. Phylogenetic tree analysis ( Figure 1) showed that the majority of the nucleotide sequences had homology similarity to genotype A at 73% (N=19/26) and 24% (N=7/26) as genotypes B, C, D, E, F and G. Genotype A reference sequences with similarity to the study sequences include: (MF169827.1 to Q9P7; AY233274.1 and AY233277.1 to Q28P7; KX520697.1 to Q4P7 and Q7P7; AY233277.1 to Q18P7, Q26P7, Q19P7, Q29P7, Q27P7, Q39P7, Q24P7, Q21P7, Q5P7, Q8P7, Q22P7, Q10P7, Q20P7, Q23P7, Q13P7). The sequences at the bottom of the tree Q6P7, Q17P7, and Q42P7 had similarity to genotype H, D, B, and E (EU694179.1, FN821495.1, AB776909.1, FN821483.1). The top sequences Q11P8, Q43P8, Q44P7, Q45P7 showed similarity to genotype H, F, G, and B (AB56246.1, AB625347.1, AB625346.1, AB562444.1, AB562456.1 and AB562461.1) as depicted in Figure 1. Sub-genotypes of the sequences were confirmed by depositing nucleotides sequences into the Genotype2pheno database. The results retrieved from the Geno2Pheno database had a 96.85% - 99.0% percentage of similarity to sub-genotype A1 for the genotype A sequences only.

### Mutations within the surface region

The prevalence of mutations in the surface gene was 47% (N=18/38) and mutations were found in the “α”, “β, “T” and outside the “α” epitope as shown in Table 2. The most common mutations on the surface region and their frequencies were S207N at 71% (27/38), followed by L216V and A194V at 23%, and the least being S204R, S117N, T143S, G145R, Y206H, P127T, Y200T, F129T and K122R all at 3% as ahown in Table 2.

**Table 2.** Frequency of the mutation within the surface region.

Epitope	Mutation	Frequency (%)
“α” epitope	K122R	3
	F134L	5
	S117N	3
	T143S	3
“β”-cell epitope	S207N	71
	Y200T	3
	G145A	3
T-helper epitope	P127T	3
Outside “α”	E164D	5
	L209V	18
	Y206H	3
	L216V	23
	A194V	23
	P70H	21
	P127L	8
	T189I	5
	S204R	3
	F129T	3

***Mutations within the polymerase region***

The prevalence of mutations in the RT of polymerase was reported at 36% (N=14/38). Mutations showed resistance to lamivudine (LMV) at (35% [5/14]), telbivudine (LdT) at (29% [4/14]), (14% [2/14]) for entecavir (ETV) and (21% [3/14]) for adefovir (ADV). Mutations causing resistance to LMV and LdT were M204V, L180M, V163I, and S202K. The S202K mutation causes resistance to ETV in addition to V204I and S169T. The ADV resistance mutations were I253Y, A236T and M250I in addition to complementary resistant mutation (L80I) not shown in the table. Multiple DRMs within a single sample were identified in one sample containing L180M, M204V, S202K and M250I mutations ( Table 3).

**Table 3.** Distribution of amino acids substitution in reverse transcriptase region of the polymerase.

Amino acids substitutions	Frequency
M129L	84%
M204V	3%
L180M	3%
V163I	78%
V173L	3%
A236T	16%
Q125E	13%
S202K	3%
L217R	21%
V214A	3%
V204I	3%
I253Y/M205I	50%
L181T/L80V	8%
L80V	40%

***Association of mutations on the surface and polymerase regions***

The prevalence of the mutations on the sequences of surface protein region was (N=18/38) and RT mutations were (N=14/38). The mean and standard deviation of the SHB mutations was 7.664, for RT mutations the mean and standard deviation was 11.214. The correlation test exhibited a weak statistical association between SHB and RT mutations (0.877). There was no statistical significance association between the SHB and RT mutations at P > 0.005.

## Discussion

HBV continues to be endemic in South Africa ( Maepa *et al*., 2022). Many studies have been conducted in this country on molecular characteristics of circulating HBV genotypes and subtypes; however, there are still areas in our country with limited published data on circulating genotypes. Therefore, this study should be considered to determine the HBV genotype and mutations circulating in HIV-infected people from KZN. The partially overlapping surface/polymerase gene region was successfully amplified in 78% (41/50), confirming the active replication of HBV and HBV/HIV co-infection in the input study samples. The phylogenetic analysis identified most of the study sequences as HBV genotype A, followed by fewer sequences showing similar homology to genotype B, C, D, E, F and G. The predominance of HBV genotype A over other genotypes is consistent with previous studies in South Africa ( Bowyer *et al*., 1997; Kimbi *et al*., 2004; Kramvis, 2014; Kramvis *et al*., 2005; Kramvis & Kew, 2007; Selabe *et al*., 2009). The predominance of HBV genotype A in HIV-infected people is consistent with another South African study, whereby genotype A was found to be the most prevalent among the antiretroviral therapy (ART) naive HIV-infected people in South Africa ( Audsley *et al*., 2010). Geno2Pheno analysis showed that majority of the sequences belonged to genotype A and sub-genotype A1. The predominance of HBV genotype A and sub-genotype A1 in our study indicates a less occurrence of genetic diversity and suggests that these strains are circulating in this geographic location due to the substandard immigration of new strains into the study region ( Kramvis *et al*., 2008; Kramvis & Kew, 2007). We determined the mutations prevalence and molecular characteristics of HBV. A total of 47% variants were observed from all sequences at different locations within the surface region (upstream and downstream of MHR). We identified K122R and T143S which are associated with diagnostic failure. Also we found G145R, P127T, S207N, Y200T, E164D, Y206H and L209V which are vaccine escape mutations, and the findings correlate to previous studies ( El-Mokhtar *et al*., 2020; Ireland *et al*., 2000; Mokaya *et al*., 2018). Contrary some of the mutations have been previously reported to have dual function as diagnostic failure and vaccine escape such as the G145R and P127T ( Archampong *et al*., 2019; Colagrossi *et al*., 2018; Kuzin *et al*., 2012; Mokaya *et al*., 2018; Yan *et al*., 2017; Yousif *et al*., 2014). We noted some mutations on the upstream position (< aa147) of the S region such as F129T and P70H but their functions have not been elucidated or reported *in vitro.* Other mutations were identified downstream position (aa147 to aa216) including: T189I, A194V, S207N, Y200T, Y206H, L209V, L216V and S204R. These mutations were previously linked with vaccination escape diagnostic failure (Colagrossi *et al*., 2018). In addition to these mutations causing vaccination escape and diagnostic failure, some mutations were reported to introduce variation in the overlapping polymerase that may cause resistance to the drugs ( Bi & Tong, 2018). The G145R and E164D mutations either occurring alone or in combination have shown to result from a substitution change in the overlapping polymerase region caused by the mutation M204V, M204I, and V173L which cause lamivudine resistance ( Colagrossi *et al*., 2018; Torresi, 2002). Variants identified in the RT region of the polymerase included drug resistance and complementary mutations. The frequency of DRMs in the RT region was 50% (N=7/14). M204V, L180M, V163I, and S202K mutations are linked to LMV and LdT resistance; S202K in addition confer resistance to EFV together with V204I. ADV resistance mutations included the I253Y, A236T, and complementary M250I. A single sample sequence contained a combination of L180M, M204V, S202K, and M250I mutations that showed to be associated with high resistance to LMV, LdT, EFV and ADV drugs. This is not opposing to previous studies whereby a combination of rtV173L + rtL180M + rtM204V was reported to confer increased resistance to LMV, LdT, and EFV ( Anastasiou *et al*., 2017). Mokaya *et al*. (2018) reported a combination of L180M + M204V + V173L + S202K + M250I to be the common cause of LMV resistance in South Africa. Contrary, the presence of multiple mutations combination may also induce cross-resistance to other drugs. Both L180M and M204V increased cross-resistance to other drugs and decreased sensitivity to ETV ( Sheldon *et al*., 2005). On the contrary, these combined L180M + M204V + S202K + M250I and L180M + M204V + V173L + S202K + M250I mutations are reported to increase susceptibility to ADV due to the presence of the secondary mutation V173L which is missing from our combination of DRMs. However, the in vitro functional studies on drug resistance and clinical implications of the combined mutations L180M + M204V + S202K + M250I have not been established but we suggest they could have implication on the treatment of HBV in HIV-coinfected people. The following compensatory mutations S202K, Q125E, L217R, V124A, V204I, and T128A were identified, and other identified DRMs were T189I, Y206H, L216V, and S209N. Functions of these mutations on drug resistance are limited and more studies are needed to understand their functions. The detection of DRMs in the sequences of patients with no previous experience with ART suggests that the treatment-naive patients might be infected by a mutant virus derived from a treated patient who was failing treatment ( Tan *et al*., 2012; Zhao *et al*., 2016). This finding agrees with a previous study that reported naturally occurring RT mutations associated with HBV drug resistance from treatment naive with HBV/HIV coinfection patients in China ( Tan *et al*., 2012; Vutien *et al*., 2014; Zhang *et al*., 2016). From South Africa, the M204I DRMs were the commonly identified in therapy-naive individuals who are co-infected with HBV/HIV ( Selabe *et al*., 2007). As this study did not find mutations linked to resistance to tenofovir, because it has a high genetic barrier to resistance and maintains effective suppression of HBV in mono-infected and HBV/HIV co-infected individuals. There was no statistical significance between S and RT mutations at P > 0.005 and there is no association between RT and SHB mutations indicated by a weak at 0.877 correlation test.

The findings of the study should be considered, bearing in mind limitations, including the design of a cross-sectional study with a small sample size. The genotyping of the HBV was not based on full genome sequences which could have identified clearly the mixed base clustering of the sequences to the genotypes. Despite this, the study provides crucial evidence for the limited knowledge of circulating HBV genotypes and mutations in HIV-infected people. The results encourage a larger sample size to provide a true representative of dominant mutations in populations, which improves the generalization of the results.

## Conclusion

This study reports on the prevalence of HBV genotype A among HIV-infected patients. Furthermore, it provides evidence on the presence of HBV mutations related to immune escape and drug resistance in people co-infected with HBV/HIV in KwaZulu-Natal, South Africa. These mutations may have clinical implications for the diagnosis and treatment of HBV in HBV/HIV co-infected individuals. We suggest that these mutations could have therapeutic consequences by influencing the correct treatment of HBV in people co-infected with HBV/HIV requiring closer attention to screening for HBV mutations in HIV-infected people. Additional *in vitro* studies are required to investigate how immune escape and drug resistance mutations affect the diagnosis and treatment of HBV in individuals co-infected with HBV/HIV, and *in vitro* studies may yield results that are more conclusive on the clinical implications of these mutations on the diagnosis and treatment of HBV in people co-infected with HBV/HIV.

## Data Availability

Figshare: data set for patients’ demographics, HIV and HBV test 2017-2019.xlsx, https://doi.org/10.6084/m9.figshare.23946621.v1 ( Modise, 2023a). Figshare: PCR amplicon gel electrophoresis of HBV overlapping surface region, https://doi.org/10.6084/m9.figshare.23815278.v1 ( Modise, 2023b). Figshare: PCR amplicon gel image of HBV overlapping surface region.pdf, https://doi.org/10.6084/m9.figshare.23946639.v1 ( Modise, 2023c). Figshare: Reference sequences accession number and genotype, https://doi.org/10.6084/m9.figshare.23946642.v1 ( Modise, 2023d). Data are available under the terms of the Creative Commons Attribution 4.0 International license (CC-BY 4.0).
