# Investigation of mutations in the partial sequences of the surface and polymerase genes of hepatitis B virus associated with immune escape and drug resistance in HIV-infected patients

**DOI:** 10.12688/f1000research.132498.4

**Published:** 2025-08-21

**Authors:** Lorato Modise, Nomathamsanqa Sithebe, Hazel Mufhandu

**Affiliations:** 1 Biological Sciences, North West University, Mahikeng, North West, 2735, South Africa

**Keywords:** Hepatitis B virus, HIV, Drug resistance, Vaccine escape, Mutation, Co-infection

## Abstract

**Background:**

Co-infection of hepatitis B virus (HBV) and human immunodeficiency virus (HIV) has an impact on high HBV replication and progression to liver cancer. These may lead to cross-resistance of drugs due to natural mutations or therapeutic pressure. These require continuous monitoring of HBV variants for better diagnosis and treatment strategies.

**Methods:**

Convenience sampling was used to collect fifty archival sera from Inkosi Albert Luthuli Central Hospital. Sera were subjected to HBsAg screening using ELISA, DNA extraction, PCR amplification, Sanger sequencing, genotype prediction and mutation analysis.

**Results:**

Of the 50 samples, 86% (43/50) were HBsAg positive; 82% (41/50) PCR positive with 92% (38/41) sequenced and only 26 sequences were subjected to molecular characterization. The HBV sequences showed similarity to genotype A (73% [19/26]), genotypes G (5% [3/26]) and genotype C (15% [4/26]). Prevalence of the mutations in the surface region was (47% [18/38]); including diagnostic failure (K122R and T143S) and immune escape mutations (P127T, G145R, S207N, Y200T, E164D, Y206H and L209V). The mutations in the RT were at (36% [14/38]) with drug resistance mutations (DRM) at (50% [7/14]). Mutations showed resistance to lamivudine (LMV) at (35% [5/14]), telbivudine (LdT) at (29% [4/14]), (14% [2/14]) for entecavir (ETV) and (21% [3/14]) for adefovir (ADV). One sample had a combination of L180M, M204V, S202K, and M250I mutations.

**Conclusions:**

Our findings highlight the prevalence of HBV genotype A in HIV-infected patients in South Africa. The study provides evidence of mutations linked to immune evasion and drug resistance; this infers that these mutations may have clinical implications for the diagnosis and treatment of HBV in HBV/HIV co-infected individuals. Further *in vitro* studies must be conducted to explore the impact of the identified mutation on the surface protein expression during diagnosis; phenotype impact of the mutant virus towards the antiviral drugs.

## Introduction

The *Orthohepadnavirus* genus is a significant group of human viruses, and the HBV is a prototype of the *Hepadnaviridae* family ( Summers *et al.*, 1975). More than 300 million people worldwide are chronically infected with hepatitis B, which can lead to severe liver disease and hepatocellular carcinoma (HCC), which accounts for more than 1 million annual deaths ( Musyoki *et al.*, 2015). HBV/HIV co-infections in the region of South Africa (SA) are estimated to range from 5% to 23% ( Anastasiou *et al*., 2017; Kaswa & de Villiers, 2023; King & Hagemeister, 2016). However, only limited evidence is available for co-infection in KwaZulu-Natal (KZN) province with a hepatitis B surface antigen (HBsAg) seroprevalence of 8.5% reported in HIV-infected people ( Msomi *et al*., 2020). The management of Hepatitis B include prevention by vaccination and treatment using the antiviral drugs. The introduction of the hepatitis B vaccine into the South African Expanded Program of Immunization (EPI) in 1995 led to a reduction in liver diseases ( Spearman *et al*., 2013). Treatment for co-infection with HBV/HIV uses drugs that are effective against both HIV and HBV including, a combination of tenofovir/(TDF) and lamivudine (LMV)/emtricitabine (FTC) /efavirenz (EFV) as outlined by Maponga *et al*. (2020). However, HBV vaccination and treatment may be threatened by the appearance of mutations on the HBV genome that could cause unfavourable clinical effects, such as vaccine escape and drug resistance. The HBV genome features are characterized by overlapping genes, notably the surface (S) gene within the polymerase (pol) gene. The S gene encodes the surface antigen (HBsAg), contains the major hydrophilic region (MHR) along with ‘α’ determinant (aa124-aa147) region, which is crucial for immune recognition and diagnostic purposes ( Liu *et al*., 2017). This overlap causes mutations in one gene to affect the other, impacting HBV evolution, replication, antiviral effectiveness, and immune escape. Mutations in the ‘α’ determinant hinder anti-HBs binding and can lead to the emergence of drug resistance mutations (DRM) in the YMDD motif of pol region ( Adesina *et al*., 2021; Cooreman *et al*., 2001). Moreover, changes near the YMDD motif contribute to drug resistance in treatments such as lamivudine, adefovir, and entecavir. Several studies have revealed M204V/I in the YMDD motif (amino acid 203±206) among HIV-infected drug-naive and drug-experienced people who received long term LMV therapy ( Mokaya *et al*., 2018; Selabe *et al*., 2007; Selabe *et al*., 2009). Also, previous studies showed drug resistance resulting from a single or combination of the following mutations: V173L, L180M, M204V/I, and A181V/T to lamivudine, rtA181V/T and rtN236T to adefovir, or rtI169T, rtI184G, rtS202G/I and rtM250V to entecavir, in treatment experienced and treatment naïve people ( Colagrossi *et al*., 2018; Lai *et al*., 2003; Torresi, 2002; Zöllner *et al*., 2001). Mutations in the pol gene also impact the surface gene with the previously identified DRM in pol leading to the emergence of immune escape mutations in the MHR ( Torresi, 2002). The prevalence of HBV has been documented in several studies among HIV positive people in KZN ( Millar *et al*., 2023; Samsunder *et al*., 2019). However, investigations on the HBV genotype, mutations associated with immune escape and drug resistance are still scarce in this region, and most studies have focused on reporting seroprevalence. The aim of the study was to describe the prevalence and molecular characterization of HBV mutations associated with immune escape and drug resistance in HIV-infected individuals in Durban, KZN, South Africa.

## Methods

### Study design and population

We applied a convenience sampling method to collect fifty archival sera in this descriptive exploratory investigation. The sample size was not determined, and we used samples that were already available. Sera were from people who underwent HIV testing at the Inkosi Albert Luthuli Central Hospital (NHLS-IALCH-NHLS) in Durban, KwaZulu-Natal Province, South Africa. Based on the sample size of 50 sera, we used the confidence level of 95% and the margin of error for our study was 14%. The 50 participants included men and women who previously tested positive for HBsAg and HIV. Participants received a written informed consent form, the information on the consent form was given in a language the patient understands, which is english and their native language (Zulu). Demographic data (age, sex, and ethnicity) were also collected. The specimen’s codes were de-linked to keep patients anonymous; with only additional data on the age and gender of the study participants provided.

### Laboratory testing

***Hepatitis B surface antigen (HBsAg) assay***

The Monolisa HBsAg ultra-confirmatory kit was used in according to the manufacturer’s instructions to perform an enzyme-linked immunosorbent assay (ELISA) on samples previously positive for HBsAg to confirm the presence of HBsAg marker (BioRad, Raymond Poincare, Marnes-la-Coquette, France). To identify and measure the presence of HBsAg, excess antibodies (anti-HBs; anti-HBs diluent: neutralization reagent) were used to neutralize 200 μL of undiluted sera specimens. At 450 nm, the optical density (OD) index of the sample was determined and compared to the cut-off value (COV) mean of a negative control. Reactive specimens for HBsAg were defined as those with an index greater than or equal to the COV.

***DNA extraction of HBV***

The sera obtained from patients were used to extract HBV deoxyribonucleic acid (DNA) using the QIAamp DNA Mini kit (catalog number: 51304) from (Qiagen, Hilden, Germany) following the manufacturer’s instructions. This technique allows the isolation and purification of total DNA from contaminants, inhibitors, and nucleases in the serum. An aliquot of 200 μL of the serum was added into 1.5 μL Eppendorf tube, to which 20 μL of proteinase K and 200 μL buffer AL (binding buffer mixed with poly [A] carrier RNA) was added. The mixture was pulse-vortex for 15 seconds to allow lysis of the mixture and destroy RNA, followed by a 10-minute incubation at 56 °C. The mixture was then transferred to a QIAamp spin column to allow binding of the DNA and centrifuged for 1 minute at 8 000 rpm. The column was placed into a clean collection tube, then 500 μL of buffer AW1 was added, and it was centrifuged for 1 minute at 8 000 rpm. The solution was aspirated, 500 μL of buffer AW2 was added to purify the DNA, and it was followed by centrifugation for 3 minutes at 14 000 rpm. The QIAamp spin column was placed in a sterile 1.5 μL Eppendorf tube, and 50 μL of elution buffer (provided by the kit as buffer AE) was added directly into the column and incubated at room temperature for 5 minutes to precipitate the DNA. The DNA was eluted by centrifugation at 8 000 rpm for 1 minute and stored at -20 °C until further analyses were performed. The negative control, consisting of nuclease-free water, was included in the extraction procedure to identify contamination.

### Polymerase chain reaction

***First round and nested-PCR***

A nested polymerase chain reaction (PCR) amplification of the overlapping surface/polymerase gene covering nucleotides 256 to 796 from *EcoRI* site was done as described previously ( Mphahlele, 2008) with slight modification. Outer sense strand (forward primer) S1 (5′-CCT GCT GGT GGC TCC AGT TC-3′), and anti-sense strand (reverse primer) S2Na (5′-CCA CAA TTC KTTGAC ATA CTT TCC A-3′) were used. The master mix were prepared using Ampli *Taq* Gold DNA polymerase (ThermoFisher Scientific, Waltham, Massachusetts, United States). For each sample the following reagent volumes and concentration of the master mix were prepared as follows: 18.5 μL nuclease-free water, 2.5 μL of 1x PCR buffer with MgCl _2_, 0.5 μL (0.2 mM dNTP mix), 0.5 μL (10 μM) forward primer S1; 0.5 μL (10 μM) reverse S2Na anti-sense primer, 0.125 *Taq* DNA polymerase. A total of 22.1 μL of master mix was aliquoted into a 0.5 mL thin-walled PCR tube and 3 μL of DNA template was added. The PCR reaction mixtures (25.1 μL) was subjected to amplification of HBV DNA, carried out in an automated touch down thermal cycler CFX96 (Bio-Rad, Raymond Poincare, Marnes-la-Coquette, France). The HBV DNA amplification conditions were initial denaturation at 95 °C for 4 minutes, followed by 40× cycles involving denaturation at 95 °C for 4 minutes, annealing at 58 °C for 30 seconds, elongation at 72 °C for 1 minute, and final extension at 72 °C for 10 minutes.

***Nested PCR***

First round PCR product was used as a template for nested PCR. An aliquot of 3 μL of the first round PCR reaction was subjected to a nested PCR, the master mix volume and concentration were prepared as same for the first round PCR. The nested PCR conditions used were the same as first round PCR protocol except the annealing temperature at 55 °C for 45 seconds. Forward primers S6E (5′-GAGAAT TCCGAGGACTGG GGA CCC TG-3′) and reverse primer S7B (5′-CGG GAT CCT TAG GGT TTA AAT GTATAC C-3′) were used during nested PCR. The negative control consisting of nuclease-free water and a positive control were included in the PCR amplification assays.

***PCR products verification***

PCR amplification products were verified using 1% agarose gel (ThermoFischer, Waltham, Massachusetts) stained with 0.15 U/μL ethidium bromide (Biorad, California, USA). Aliquot of 10 μL PCR amplicon product was mixed with 2 μL 10x loading buffer (ThermoFischer, Waltham, Massachusetts). The mixtures were run on 1% gel along with a 1 Kb Invitrogen molecular weight maker (ThermoFischer, Waltham, Massachusetts) as a band size reference. The agarose gel was run at 100 V for 45 minutes. The gel was placed inside the ultraviolet (UV) transilluminator (Bio-rad, Hercules, California, United States) to visualise and image capturing.

***Sequencing reaction***

The PCR products and the nested PCR primers S6E and S7B were sent for bi-directional Sanger sequencing at the Inqaba (Inqaba Biotechnological Industry, Pretoria, South Africa). The amplicons were prepared for direct sequencing using the BigDye terminator v3.0 cycle sequencing ready reaction kit (catalog number: 4458687) from (ThermoFischer, Waltham, Massachusetts). Briefly, an aliquot of 50 μL of the 1:1 ratio of sodium acetate: ethanol (NaAc:EtOH) was added to the amplicons solution and centrifuged at 2000 g for 30 minutes. The well plates were inverted and centrifuged at 150 g for 1 minute. Pre-chilled 70% ethanol was added into the wells and then centrifuged at 2000 g for 5 minutes. The pellets were dried at 65 °C for 5 minutes followed by an addition of 10 μL Hi-Di formamide for 5 minutes and loaded into the sequencing machine ABI 3130XL genetic analyser (Applied Biosystems, Foster City, CA).

### Sequences analysis

Nucleotide sequences of HBV chromatograms were viewed and edited by removing unwanted and mixed nucleotides character from the sequences by ChromasPro, version.1. The contiguous sequences were formed by joining overlapping DNA sequences of a gene using BioEdit. The consensus sequences were compared with the GenBank complementary genotype sequences using the basic local alignment search tool (BLAST). Representative sequences belonging to different genotypes were redeemed from GenBank to make comparisons with the study sequences. Multiple sequences alignment was performed with ClustalW within the MEGA software package version 7.0 (TomHall, North Carolina State University). Genotyping and identification of the sequence was done on Geno2pheno ( Geno2pheno, 2023). Sequences were uploaded into the website where they were aligned at the sequence position 1 to 3182 (~3.18 kbp) relative to the Hepatitis B virus (taxon:10407) and a boostscan analysis of ≥ 80.0 was considered positive.

### Mutations analysis

The aligned sequences were uploaded into the BioEdit and analysed for nucleotide base and amino acids changes. The sequences were further uploaded into the Geno2pheno to identify DRMs in the reverse transcriptase (RT) of polymerase and mutations in the overlapping S region.

### Statistical analyses

Microsoft Excel and the data science statistical program STATA were used for data analyses (version 15). Excel (.csv.) file was imported into STATA and was used to calculate the frequency of age as numeric values and the frequency of HBsAg and mutations as categorical and numeric variables. The proportions of data reported as medians with interquartile ranges (IQR) for continuous variables (age) as frequencies and percentages for categorical variables (sex, ethnicity and mutations).

### Ethical approval

The study ethics certificate was provided by the North-West University research ethics regulatory committee (ref. NWU-00068-15-A9).

## Results

### Baseline demographic data

The baseline demographics of the study showed that all 50 HIV positive samples included were female at 64% (n=32/50) and 36% (n=18/50) male as shown in Table 1. The median age was 33 years [IQR: 18-55] and there was no statistical difference on age between the female and male black Africans at p=0.8.

**Table 1.** Demographics data of the patients by age, sex, HBV and HIV antigens markers.

Age in years (median, IQR)	Sex		HIV p24 antigen		HBsAg antigen	
[%; n/50] 33[9.25,2736.25]	Female	Male	HIV (+) Female	HIV (+) Male	HBsAg (+) Female	HBsAg (+) Male
18-35	59% (n=19)	66% (n=12)	40% (n=20)	14% (n=7)	34% (n=17)	12% (n=6)
36-55	41% (n=13)	33% (n=6)	12% (n=12)	22% (n=11)	24% (n=12)	16% (n=8)
Total	64% (n=32)	36% (n=18)	64% (n=32)	36% (n=18)	58% (n=29)	28% (n=14)
*p-value*	0.8		0.1		0.6	

### Laboratory testing

***HBsAg assay***

The confirmatory screening of HBsAg reported a prevalence of 86% (n=43/50) with 12% (n=6/50) being negative and 2% (n=1/50) missing data ( Table 1). Majority of the female’s participant were HBsAg positive at 58% (n=29/50) when compared to the males at 28% (n=14/50) as shown in Table 1.

***PCR amplification and genotyping***

The PCR amplification of HBV DNA amplicons was successful in 82% (n=41/50) of the samples and consisting of overlapping surface/polymerase region. PCR amplification could not be obtained for 18% (n=09/50) samples. Only 92% (n=38/41) sequence products could be obtained by Sanger sequencing with only 26 sequences used to perform genotyping and 12 sequences were excluded due to the poor quality of the sequence reads. The genotypes of the sequences were confirmed by depositing nucleotides sequences into the Genotype2pheno database which showed that most of the nucleotide sequences had homology similarity to genotype A at 73% (n=19/26) and 12% (n=3/26) as genotype G and 15% (n=4/26) being genotype C ( Table 2). The study sequences with the highest similarity identity to genotype A were: (Q4P7 and Q7P7 to KX520697.1|South Africa; Q9P7 to U87728.1|South Africa; Q27P7 to AY233274.1|South Africa. Reference sequence of AY233277.1|South Africa had between 97%-99% similarity to fifteen sequences (Q5P7, Q8P7, Q10P7, Q13P7, Q18P7, Q19P7, Q20P7, Q21P7, Q22P7, Q23P7, Q24P7, Q26P7, Q27P7, Q29P7 and Q39P7). The sequences Q6P7, Q17P7, and Q42P7 had similarity to genotype G (EU694179.1|South Africa and sequences Q11P7, Q43P7, Q44P7, Q45P7 showed similarity to genotype C AB562444.1|Vietnam ( Table 2). Sub-genotypes of the sequences were confirmed by depositing nucleotides sequences into the Genotype2pheno database. The results retrieved from the Geno2Pheno database had a 96.85%-99.0% percentage of similarity to sub-genotype A1 for the genotype A sequences only.

**Table 2.** Genotype identity of the study sequences by comparing with standard representative sequences obtained from GenBank.

Sample code	Reference accession no ^1^	Genotype identity	Homology similarity (%)
Q4P7	KX520697.1|South Africa	A	99.46
Q5P7	AY233277.1|South Africa	A	98.96
Q6P7	EU694179.1|South Africa	G	99.83
Q7P7	KX520697.1|South Africa	A	98.76
Q8P7	AY233277.1|South Africa	A	98.42
Q9P7	U87728.1|South Africa	A	98.98
Q10P7	AY233277.1|South Africa	A	97.62
Q11P7	AB562444.1|Vietnam	C	99.39
Q13P7	AY233277.1|South Africa	A	97.95
Q17P7	EU694179.1|South Africa	G	99.32
Q18P7	AY233277.1|South Africa	A	98.56
Q19P7	AY233277.1|South Africa	A	99.18
Q20P7	AY233277.1|South Africa	A	99.78
Q21P7	AY233277.1|South Africa	A	99.96
Q22P7	AY233277.1|South Africa	A	98.49
Q23P7	AY233277.1|South Africa	A	99.28
Q24P7	AY233277.1|South Africa	A	99.94
Q26P7	AY233277.1|South Africa	A	97.96
Q27P7	AY233274.1|South Africa	A	99.08
Q29P7	AY233277.1|South Africa	A	99.20
Q39P7	AY233277.1|South Africa	A	97.50
Q42P7	EU694179.1|South Africa	G	98.82
Q43P7	AB562444.1|Vietnam	C	99.28
Q44P7	AB562444.1|Vietnam	C	97.18
Q45P7	AB562444.1|Vietnam	C	99.42

^1^ Reference sequences obtained from GenBank (designated by accession numbers). The study sequences are represented by the letters Q, P and are followed by numeric values.

### Mutations within the surface region

The prevalence of mutations in the surface gene was 47% (n=18/38) and mutations were found in the “α”, “β”, “T” and outside the “α” epitope as shown in Table 3. The most common mutations on the surface region and their frequencies were S207N at 71% (27/38), followed by L216V and A194V at 23%, and the least prevalent being S204R, S117N, T143S, G145R, Y206H, P127T, Y200T, F129T and K122R all at 3% as shown in Table 3.

**Table 3.** Amino acids substitution in surface, polymerase and their associated functions.

Surface region				Reverse transcriptase	
Epitope	Mutation	Frequency	Function	Mutation	Drugs of resistance
“α” epitope	K122R	3	Diagnostic failure	M129L	Compensatory/epistasis to LMV
	F134L	5	Diagnostic failure	M204V	LMV, LdT
	S117N	3	Diagnostic failure	L180M	LMV, LdT
	T143S	3	Diagnostic failure	V163I	LMV, LdT
“β” epitope	S207N	71	Vaccine escape	V173L	LMV
	Y200T	3	Vaccine escape	A236T	ADV
	G145R	3	Vaccine escape Diagnostic failure	Q125E	Compensatory/epistasis to LMV
T-helper epitope	P127T	3	Vaccine escape Diagnostic failure	S202K	LMV, LdT, ETV
Outside “α”	E164D	5	Vaccine escape	L217R	Compensatory/epistasis to LMV
	L209V	18	Vaccine escape/ cause RT mutations (M204V/M204I/V173L)	V214A	Compensatory/epistasis to LMV
	Y206H	3	Vaccine escape	V204I, S169T	LMV, ETV
	L216V	23	Vaccine escape	I253Y/M205I	ADV
	A194V	23	Vaccine escape	L181T/L80V	LMV,
	P70H	21	Unknown	L80V	LMV
	P127L	8	Vaccine escape	L180M+M204V+S202K+ M250I	LMV+LdT+ADV and EFV
	T189I	5	Vaccine escape		
	S204R	3	Vaccine escape		
	F129T	3	Unknown		

***Mutations within the polymerase region***

The prevalence of mutations in the RT of polymerase was reported at 36% (n=14/38). Mutations showed resistance to lamivudine (LMV) at (35% [5/14]), telbivudine (LdT) at (29% [4/14]), (14% [2/14]) for entecavir (ETV) and (21% [3/14]) for adefovir (ADV). Mutations causing resistance to LMV and LdT were M204V, L180M, V163I, and S202K. The S202K mutation causes resistance to ETV in addition to V204I and S169T. The ADV resistance mutations were I253Y, A236T and M250I in addition to complementary resistant mutation (L80I) not shown in the table. Multiple DRMs within a single sample were identified in one sample containing L180M, M204V, S202K and M250I mutations ( Table 3).

## Discussion

HBV continues to be endemic in South Africa ( Maepa *et al*., 2022). However, there are still areas in our country with limited published data on circulating genotypes. Therefore, this study considered to determine the HBV genotype and mutations circulating in HIV-infected people from KZN. The partially overlapping surface/polymerase gene region was successfully amplified in 78% (41/50), confirming the active replication of HBV in HIV co-infected people. Geno2pheno characterized majority of the sequences as HBV genotype A, followed by fewer sequences showing similar homology to genotype C and G. The predominance of HBV genotype A over other genotypes is consistent with previous studies in South Africa ( Bowyer *et al*., 1997; Kimbi *et al*., 2004; Kramvis, 2014; Kramvis *et al*., 2005; Kramvis & Kew, 2007; Selabe *et al*., 2009). Genotype A was found to be the most prevalent among the antiretroviral therapy (ART) naive HIV-infected people in South Africa ( Audsley *et al*., 2010). This suggest that the mutant HBV is common in both naive and experience HIV positive people. The genotype A mutant virus may have a high transmission rate and low response to ART by an individual. The uncommon genotype G showed similarity to a reference sequence from South Africa with HIV co-infection ( Lukhwareni *et al*., 2020). The occurrence of genotype C may be due to migration into South Africa. The predominance of HBV genotype A in our study indicates a less occurrence of genetic diversity and suggests that these strains are circulating in this geographic location due to the substandard immigration of new strains into the study region ( Kramvis *et al*., 2008; Kramvis & Kew, 2007). A total of 47% variants were observed from all sequences at different locations within the surface region (upstream and downstream of MHR). We identified K122R and T143S which are characterized to be associated with diagnostic failure. Also, we found G145R, P127T, S207N, Y200T, E164D, Y206H and L209V which are immune escape mutations, and the findings correlate to previous studies ( El-Mokhtar *et al*., 2020; Ireland *et al*., 2000; Mokaya *et al*., 2018). Contrary some of the mutations have been previously reported to have dual function as diagnostic failure and immune escape such as the G145R and P127T ( Archampong *et al*., 2019; Colagrossi *et al*., 2018; Kuzin *et al*., 2012; Mokaya *et al*., 2018; Yan *et al*., 2017; Yousif *et al*., 2014). We identified some mutations on the upstream position (< aa147) of the S region such as F129T and P70H but their functions have not been elucidated or reported *in vitro.* Other mutations were identified downstream position >aa147 (to aa216) including: T189I, A194V, S207N, Y200T, Y206H, L209V, L216V and S204R. These mutations were previously linked with vaccination escape diagnostic failure ( Colagrossi *et al*., 2018). The G145R and E164D mutations either occurring alone or in combination have shown to result from a substitution change in the overlapping polymerase region caused by the mutation M204V, M204I, and V173L which cause lamivudine resistance ( Colagrossi *et al*., 2018; Torresi, 2002). The frequency of DRMs in the RT region was 50% (n=7/14). M204V, L180M, V163I, and S202K mutations are linked to LMV and LdT resistance; S202K in addition confer resistance to EFV together with V204I. ADV resistance mutations included the I253Y, A236T, and complementary M250I. A single sample sequence contained a combination of L180M, M204V, S202K, and M250I mutations that showed to be associated with high resistance to LMV, LdT, EFV and ADV drugs. This is not opposing to previous studies on combination of DRMs ( Anastasiou *et al*., 2017; Mokaya *et al*., 2018). Contrary, the presence of multiple mutations combination may also induce cross-resistance to other drugs. Both L180M and M204V increased cross-resistance to other drugs and decreased sensitivity to ETV ( Sheldon *et al*., 2005). On the contrary, these combined L180M + M204V + S202K + M250I and L180M + M204V + V173L + S202K + M250I mutations are reported to increase susceptibility to ADV due to the presence of the secondary mutation V173L which is missing from our combination of DRMs. However, the *in vitro* functional studies on the combined mutations L180M + M204V + S202K + M250I have not been established but we suggest they could have implication on the treatment of HBV in HIV-coinfected people. The following compensatory mutations S202K, Q125E, L217R, V124A, V204I, and T128A were identified, and other identified DRMs were T189I, Y206H, L216V, and S209N. These mutations have effects on treatment resistance; further research is required to fully comprehend how they affect antiviral drugs. The identification of DRMs in HBV treatment-naive patient increases the possibility that the treatment-naive patient may be infected with a mutant HBV virus that was either naturally occurring resistance mutations or derived from a patient who developed resistance after antiviral treatment ( Belyhun *et al*., 2017; Tan *et al*., 2012; Vutien *et al*., 2014; Zhao *et al*., 2016). We conclude that additional research is required to identify the mechanism producing the DRMs in HIV-positive patients who have never received HBV therapy. Although they can’t extrapolate on past treatment histories, the DRMs identified in this study provide information on the present status of treatment resistance. Second, the DRMs identified in this study suggest that those with mutations resistance to ARTs may have fewer options for therapy. The study does not support whether dual HBV/HIV therapy was effective on HBV or the effect of HIV treatment on ART-naïve HBV. This does not change how the DRMs sequences results are interpreted, which highlights the need of HBV testing by genotyping in addition to HBV and HCV testing prior to starting ART in HIV patients, as recommended by WHO *et al*. (2017).

## Limitations

The findings of the study should be considered, bearing in mind limitations, including the design of a cross-sectional study with a small sample size. Genotyping of the HBV was not based on full genome sequences which could have identified clearly the mixed base clustering of the sequences to the genotypes. The results encourage a larger sample size to provide a true representative of dominant mutations in the study site and other areas, which improves the generalization of the results. There is no availability of the presumptuous treatment history, and it effect on the understanding the impact of HIV treatment on the HBV mutations profile and treatment.

## Conclusion

This study reports on the prevalence of HBV genotype A among HIV-infected patients. Furthermore, it provides evidence on the presence of HBV mutations related to immune escape and drug resistance in people co-infected with HIV in KwaZulu-Natal, South Africa. A thorough-synchronizes diagnosis and antiviral therapy for HBV patients with HIV co-infection should include proper HBV diagnosis by resistance testing to minimize the emergence and spread of antiviral drug resistance. We recommend that further *in vitro* studies be conducted to explore the impact and functions of the S and pol mutation on the protein expression and DRMs towards the antiviral resistance.

## Data Availability

Figshare: data set for patients’ demographics, HIV and HBV test 2017-2019.xlsx, https://doi.org/10.6084/m9.figshare.23946621.v1 ( Modise, 2023a). Figshare: PCR amplicon gel electrophoresis of HBV overlapping surface region, https://doi.org/10.6084/m9.figshare.23815278.v1 ( Modise, 2023b). Figshare: PCR amplicon gel image of HBV overlapping surface region.pdf, https://doi.org/10.6084/m9.figshare.23946639.v1 ( Modise, 2023c). Data are available under the terms of the Creative Commons Attribution 4.0 International license (CC-BY 4.0).
