# Supplementary material for: Investigation of mutations in the partial sequences of the surface and polymerase genes of hepatitis B virus associated with immune escape and drug resistance in HIV-infected patients
Source: F1000Res. 2025 Aug 21;12:1232. Originally published 2023 Sep 27. [Version 4] doi: 10.12688/f1000research.132498.4 (PMC12426594; doi:10.12688/f1000research.132498.4)
Supplement: Peer review history

# Reviewer report: Reviewer response for version 4

**DOI:** 10.5256/f1000research.186606.r407676

**Published:** 2025-09-11

**Authors:** Tesfaye Gelanew

**Affiliations:** 1 Armauer Hansen Research Institute, Addis Ababa, Addis Ababa, Ethiopia

I have no further comment. I endorsed the manuscript for indexing.

Is the work clearly and accurately presented and does it cite the current literature?

Yes

If applicable, is the statistical analysis and its interpretation appropriate?

Yes

Are all the source data underlying the results available to ensure full reproducibility?

Yes

Is the study design appropriate and is the work technically sound?

Yes

Are the conclusions drawn adequately supported by the results?

Yes

Are sufficient details of methods and analysis provided to allow replication by others?

Yes

Reviewer Expertise:

NA

I confirm that I have read this submission and believe that I have an appropriate level of expertise to confirm that it is of an acceptable scientific standard.

# Reviewer report: Reviewer response for version 3

**DOI:** 10.5256/f1000research.179250.r380330

**Published:** 2025-08-05

**Authors:** Nishi Prabdial-Sing

**Affiliations:** 1 Centre for Vaccines and Immunology, Division of the National Health Laboratory Service, National Institute for Communicable Diseases, Johannesburg, South Africa

Thank you to the authors for addressing some of the reviewer’s comments.

 There are still grammatical errors in the abstract.

 Abstract: Background

 ‘”cross-resistance of drugs”” what does this mean exactly, that sites on the HIV and HBV genome have shown cross-resistance of HIV antiviral drugs and to HBV antiviral drugs? I have not seen this and should be better written

 Begin the next sentence “Continuous monitoring of HBV variants is required for better…”

 Results:

 Also as you found all genotype A to be subgenotype A1, then indicate A1 in your findings and conclusions

 Genotype c, should be C

 Introduction:

 The abbreviation “DRM”, needs to be explained when used for the first time

 Method

 English should be English

 used in according should be used according

 Results:

 Mutations showed resistance to lamivudine (LMV) at (35% [5/14]), telbivudine (LdT) at (29% [4/14]), (14% [2/14]) for entecavir (ETV) and (21% [3/14]) for adefovir (ADV). Mutations causing resistance to LMV and LdT were M204V, L180M, V163I, and S202K”

 “the statement implies that the patients were treated for HBV and these DRMs were found. However, it was mentioned that these patients were Treatment naïve? Please clarify whether the patients were treatment naïve for HIV or HBV or both?

 Discussion:

 This suggest that the mutant HBV is common in both naive and

 experience HIV positive people””. This sentence is not referenced. There is only one reference for naïve, but not HIV treated persons.

 Conclusion

 thorough-synchronizes diagnosis” what does this mean?

 What do the authors mean by substandard immigration of new strains?

Is the work clearly and accurately presented and does it cite the current literature?

Yes

If applicable, is the statistical analysis and its interpretation appropriate?

I cannot comment. A qualified statistician is required.

Are all the source data underlying the results available to ensure full reproducibility?

Yes

Is the study design appropriate and is the work technically sound?

Yes

Are the conclusions drawn adequately supported by the results?

Yes

Are sufficient details of methods and analysis provided to allow replication by others?

Partly

Reviewer Expertise:

NA

I confirm that I have read this submission and believe that I have an appropriate level of expertise to confirm that it is of an acceptable scientific standard, however I have significant reservations, as outlined above.

# Reviewer report: Reviewer response for version 3

**DOI:** 10.5256/f1000research.179250.r393935

**Published:** 2025-07-28

**Authors:** Tesfaye Gelanew

**Affiliations:** 1 Armauer Hansen Research Institute, Addis Ababa, Addis Ababa, Ethiopia

The manuscript by Modise et al entitled "Investigation of Hepatitis B Virus Mutations Associated with Immune Escape and Drug Resistance in HIV-Infected Patients" is highly informative, and I concur with the authors’ observation that most studies conducted in Africa focus primarily on prevalence, while only a limited number address genotyping and drug resistance profiling. The paper is well written, with sound methodological approaches. The conclusions are appropriately toned down, clearly derived from the data, and the limitations are well articulated. I recommend acceptance in its current form. However, I have the following few minor comments.

 1. - The title lacks specificity. It should be revised to:

 Investigation of Mutations in the Partial Sequences of the S and Polymerase Genes of Hepatitis B Virus Associated with Immune Escape and Drug Resistance in HIV-Infected Patients

 2. - The introduction would benefit from expanding the brief overview of the specific genes targeted in this study—namely those encoding the surface antigen and polymerase—and their roles in immune escape and antiviral drug resistance.

Is the work clearly and accurately presented and does it cite the current literature?

Yes

If applicable, is the statistical analysis and its interpretation appropriate?

Yes

Are all the source data underlying the results available to ensure full reproducibility?

Yes

Is the study design appropriate and is the work technically sound?

Yes

Are the conclusions drawn adequately supported by the results?

Yes

Are sufficient details of methods and analysis provided to allow replication by others?

Yes

Reviewer Expertise:

My expertise is clinical microbiology and Immunology/molecular biology. My ongoing research areas are dengue virus, HBV, RSV, and pathogens causing STIs.

I confirm that I have read this submission and believe that I have an appropriate level of expertise to confirm that it is of an acceptable scientific standard, however I have significant reservations, as outlined above.

# Reviewer report: Reviewer response for version 3

**DOI:** 10.5256/f1000research.179250.r386531

**Published:** 2025-06-14

**Authors:** Anthony C. Ike

**Affiliations:** 1 University of Nigeria, Nsukka, Enugu, Nigeria

The article is well designed and carried out and was also well written or corrected to bring it to its present level. I have made few observations as listed below:

 In Page 4 under DNA extraction of HBV in Lines 4 and 10, the Eppendorf tube is 1.5 mL and not 1.5 μL.

 In the same page under Polymerase chain reaction First round and nested-PCR, in Line 8 of that section, the authors need to state whether the 0.125 of Taq DNA polymerase is the concentration (units) or volume (μL) of the enzyme.

 Under Nested PCR, the last line of the section, Line 6, the authors should state what was used as positive control and state the source if possible.  

 In page 6 under laboratory testing HBsAg assay Line 2 of the section, it should be females and not female's. Also the HBsAg positive for the females should be 91% (N=29/32) and not 58% (N=29/50), since there were only 32 females and not 50. similarly that of the males should be 78% (N=14/18) and not 28% (N=14/50), males ere 18 and not 50. This should be corrected in Table 1 and be extended to the results of HIV (+) Female and HIV (+) Male.

Is the work clearly and accurately presented and does it cite the current literature?

Yes

If applicable, is the statistical analysis and its interpretation appropriate?

Partly

Are all the source data underlying the results available to ensure full reproducibility?

Yes

Is the study design appropriate and is the work technically sound?

Yes

Are the conclusions drawn adequately supported by the results?

Yes

Are sufficient details of methods and analysis provided to allow replication by others?

Yes

Reviewer Expertise:

Medical Microbiology with Virology bias. I have researched and published works on Noroviruses, Rotaviruses, influenza virus, hepatitis B and C viruses, Dengue virus among others.

I confirm that I have read this submission and believe that I have an appropriate level of expertise to confirm that it is of an acceptable scientific standard, however I have significant reservations, as outlined above.

# Reviewer report: Reviewer response for version 2

**DOI:** 10.5256/f1000research.163315.r284916

**Published:** 2024-06-06

**Authors:** Anna McNaughton

**Affiliations:** 1 Nuffield Department of Medicine, University of Oxford Medawar Building, Oxford, England, UK

The authors have clearly revised the manuscript, taking into account some of the feedback provided previously and their metadata now looks to be more accurate. However, there are still several issues with the revised document, and particularly the analysis and presentation of the results which need correcting before the manuscript can be approved.

 HBsAg assay – COV needs defining at first use.

 Baseline demographic data – the median age is presented and the range is but I can’t see the standard deviation, despite the authors stating it in the paragraph. Table 1 should be revised to be more informative (i.e. present proportions) – if all individuals were black africans and HIV positive, this is not particularly useful information to present? I would encourage the authors to revise the data in table 1 to make it more informative, such as is there any data on their HIV treatment?

 Phylogenetic analysis – the authors state that sequences were obtained for 38/41 samples that were PCR positive, but only analysed n=26 sequences. Why were 12 sequences excluded from the analysis?

 The sequences analysed are relatively short, and as the authors state, this can affect the clustering but the phylogenetic analysis presented remains very problematic for genotyping. The reference sequences do not cluster by genotype, making it difficult to use the tree to confidently genotype the sequences presented. Whilst the authors checked a number of gtA the sequences with geno2pheno, I am puzzled by the interpretation of the other sequences (assumed not to be gtA). A number of sequences do not clearly cluster with any of the reference sequences, and the authors describe these as “24% (N=7/26) as genotypes B, C, D, E, F and G”, which is difficult to interpret. It is also a little unclear why sequences at the bottom of the tree ‘Q6P7, Q17P7, and Q42P7’ were decided to be closer to genotype H, D, B, and E than to genotype A without presenting bootstrapping data? This analysis needs revising, and perhaps the authors should consider a maximum-likelihood analysis as it may be more discriminatory for genotyping than the approach currently used. Alternatively, they may consider using geno2pheno to genotype their sequences, and present this data in a table rather than a tree?

 Association of mutations on the surface and polymerase regions – authors state the mean and standard deviation of the SHB mutations, but only provide a single number? Are the numbers presented also the number of mutations per sequence, as this is not clear?

 The discussion is too long and should be more concise – the discussion of the wider literature on drug resistant mutations could be reduced. Some context may also be useful to discuss – these samples were taken from HIV-positive individuals, so were any of them on treatment for their HIV infections and were these treatments active against HBV also? The authors mention that some patients are ART treatment naïve late in the discussion – but a little more detail on this would add to the interpretation of this study.

Is the work clearly and accurately presented and does it cite the current literature?

Partly

If applicable, is the statistical analysis and its interpretation appropriate?

Partly

Are all the source data underlying the results available to ensure full reproducibility?

Partly

Is the study design appropriate and is the work technically sound?

No

Are the conclusions drawn adequately supported by the results?

No

Are sufficient details of methods and analysis provided to allow replication by others?

Yes

Reviewer Expertise:

Virology, genomics, epidemiology, viral hepatitis

I confirm that I have read this submission and believe that I have an appropriate level of expertise to state that I do not consider it to be of an acceptable scientific standard, for reasons outlined above.

# Response

**Published:** 2025-03-12

**Authors:** Lorato Mosetsanagape Modise

**Affiliations:** Virology, Sefako Makgatho health Sciences University, Pretoria, Gauteng, South Africa

Dear reviewer

 We truly appreciate knowledge, and insightful comments on our paper. We appreciate your reviews and recommendations, which have greatly enhanced the manuscript. We have carefully considered all your comments and revised them accordingly. 

**Specific reviewer comment**

 HBsAg assay – COV needs defining at first use.

**Response**: Cut off value (COV) has been defined at first use.

**Reviewer comments**

 Baseline demographic data – the median age is presented, and the range is, but I can’t see the standard deviation, despite the authors stating it in the paragraph. Table 1 should be revised to be more informative (i.e. present proportions) – if all individuals were black Africans and HIV positive, this is not particularly useful information to present. I would encourage the authors to revise the data in table 1 to make it more informative, such as is there any data on their HIV treatment?

**Response**: 

- Since all the samples were from the same group, the information on the ethnicity of Black African has been deleted from the description in Table 1.
- The description of the statistical analysis has been revised in the text and Table 1 to be specific to the standard deviation. The median age is presented with the interquartile range (IQR) the range and did not present the standard deviation because the data in not normally distributed and there is poor statistical power. Table 1 presents the demographic data collected from the participants which includes the age, sex and test done before. The data is already presented in proportions expressed by either a count by the total number and the proportion as a percentage. The age is already presented in proportions as indicated in the text (n=19/50).
- The results are on demographic data which is the primary data on the participants HIV status. We can only report on the data that was collected and available for this study, The data on the treatment history of the participants specifically HIV treatment is not available hence, it is not included. This is highlighted in the limitations section. 

**Reviewer comments**

 Phylogenetic analysis – the authors state that sequences were obtained for 38/41 samples that were PCR positive but only analysed n=26 sequences. Why were 12 sequences excluded from the analysis? The sequences analysed are relatively short, and as the authors state, this can affect the clustering, but the phylogenetic analysis presented remains very problematic for genotyping. The reference sequences do not cluster by genotype, making it difficult to use the tree to confidently genotype the sequences presented. Whilst the authors checked a few gtA the sequences with geno2pheno, I am puzzled by the interpretation of the other sequences (assumed not to be gtA). A few sequences do not clearly cluster with any of the reference sequences, and the authors describe these as “24% (N=7/26) as genotypes B, C, D, E, F and G”, which is difficult to interpret. It is also a little unclear why sequences at the bottom of the tree ‘Q6P7, Q17P7, and Q42P7’ were decided to be closer to genotype H, D, B, and E than to genotype A without presenting bootstrapping data? This analysis needs revising, and perhaps the authors should consider a maximum-likelihood analysis as it may be more discriminatory for genotyping than the approach currently used. Alternatively, they may consider using geno2pheno to genotype their sequences,

**Response**:

- The 12 sequences excluded from the analysis had poor sequence reads on the chromatogram, and they could not form the proper consensus sequences thus they were not considered for the analysis.
- The genotyping of the sequences is revised with the use of geno2pheno software as advised and the data is presented in a table instead of a phylogenetic tree (Table 2). The sequences were uploaded onto the geno2pheno software and compared against standard (wildtype) genome with the highest nucleotide similarity to the study sequences.
- The sequences that identify as genotype A have been clarified through revision. Additionally, the 24% (N=7/26) that represent a combination of other genotypes (C and G) have been broken down to demonstrate the individual and specific genotypes C and G; 12% (N=3/26) represent genotypes G, and 15% (N=4/26) represent genotype C.

**Association of the mutations**

- Data on the association of mutations on the surface and polymerase regions has been removed due to the poor statistical power and statistical association. We focused on reporting the data on the mutations associated with drug resistance with a focus on their frequency as depicted in Table 3.

**Reviewer comment**

 The discussion is too long and should be more concise – the discussion of the wider literature on drug resistant mutations could be reduced. Some contexts may also be useful to discuss – these samples were taken from HIV-positive individuals, so were any of them on treatment for their HIV infections and were these treatments active against HBV also?

**Response**:

- The discussion has been summarised. The data on the treatment history of the participants specifically HIV treatment is not available hence, it is not included. Secondly, we don’t know if HIV treatments is active against HBV because we do not have the information currently available to support that. However, we know the treatment history of HBV and that the participants are naïve to HBV ARTs. We have also added information on the results interpretation of the mutations in HBV patients that are HBV treatment naïve (last paragraph under the discussion section).
- Results
- Table 1: The median age was 33 years [IQR: 18-55] and there was no statistical difference on age between the genders with p=0.8.
- 92% (N=38/41) sequence products could be obtained by Sanger sequencing with only 26/38 sequences used to perform genotyping, that is, 12 sequences were excluded due to the poor quality of their sequence reads.
- The genotype of the sequences was confirmed by depositing nucleotide sequences into the Genotype2pheno database, which showed that most of the nucleotide sequences had homology with genotype A at 73% (N=19/26), genotype G at 12% (N=3/26), and genotype C at 15% (N=4/26) (Table 2).
- The study sequences with the highest similarity to Genotype A were: (Q4P7 and Q7P7 to KX520697.1|South Africa; Q9P7 to U87728.1|South Africa; Q27P7 to AY233274.1|South Africa.  Reference sequence of AY233277.1|South Africa had between 97%-99% similarity to fifteen sequences (Q5P7, Q8P7, Q10P7, Q13P7, Q18P7, Q19P7, Q20P7, Q21P7, Q22P7, Q23P7, Q24P7, Q26P7, Q27P7, Q29P7 and Q39P7).  The sequences Q6P7, Q17P7 and Q42P7 had similarity to genotype G (EU694179.1|South Africa; sequences Q11P7, Q43P7, Q44P7 and Q45P7 showed similarity to genotype C AB562444.1|Vietnam (Table 2).  Sub-genotypes of the sequences were confirmed by depositing nucleotides sequences into the Genotype2pheno database. The results retrieved from the Geno2Pheno database had a 96.85%-99.0% percentage of similarity to sub-genotype A1 for the genotype A sequences only.

**Discussion**

 Discussion on the interpretation of mutations in treatment-naïve has been revised. This includes clarity on the impact of the mutations on the treatment’s status of the participants. We also discuss how the study cannot answer on whether the treatment was active against HBV (see the last paragraph under the discussion section). However, we made recommendation that further in vitro studies must investigate the mechanism of action by DRMs on ARTs in vitro assays (last sentence under conclusion).

**Additional correction and response**

 Reference removed from the list 

 1. Kimura M: A simple method for estimating evolutionary rates of base substitutions through comparative studies of nucleotide sequences.

 J. Mol. Evol. 1980; 16(2): 111–120. PubMed Abstract|Publisher Full Text.

 2. Kumar S, Tamura K, Nei M: MEGA3: Integrated software for Molecular Evolutionary Genetics Analysis and sequence alignment. Brief. Bioinform. 2004; 5(2): 150–163. PubMed Abstract|Publisher Full Text.

 3. Malik A, Singhal DK, Albanyan A, et al.: Hepatitis B virus gene mutations in liver diseases: a report from New Delhi. PLoS One. 2012; 7(6): e39028. PubMed Abstract|Publisher Full Text|Free Full Text.

 4. Saitou N, Nei M: The neighbor-joining method: a new method for reconstructing phylogenetic trees. Mol. Biol. Evol. 1987; 4(4): 406–425. .

 Updated references and citation

 1.    Belyhun, Yeshambel, Melanie Maier, and Uwe Gerd Liebert. "HIV therapy with unknown HBV status is responsible for higher rate of HBV genome variability in Ethiopia." Antiviral therapy 22.2 (2017): 97-111.

 2.    Geno2pheno. HBV genotyping 2023 [Available from: https://hbv.geno2pheno.org/.

 3.    World Health Organization. Guidelines on Hepatitis B and C Testing 2017 update (available at https://apps.who.int/iris/bitstream/handle/10665/254621/9789241549981- eng.pdf.).

# Reviewer report: Reviewer response for version 2

**DOI:** 10.5256/f1000research.163315.r284915

**Published:** 2024-06-03

**Authors:** Nishi Prabdial-Sing

**Affiliations:** 1 Centre for Vaccines and Immunology, Division of the National Health Laboratory Service, National Institute for Communicable Diseases, Johannesburg, South Africa

Thank you to the authors for addressing some of the reviewer’s comments.

 There are still grammatical errors in the abstract.

**Abstract:**

Background:

- “…cross-resistance to drugs…”, not "of drugs".
- Begin the next sentence “Continuous monitoring of HBV variants is required for better…”

Methods:

- “Sera” is in the middle of the sentence and should be “sera”.

Results:

- “Surface” is in the middle of the sentence and should be “surface”.
- Also as you found all genotype A to be subgenotype A1, then indicate A1 in your findings and conclusions.

**Introduction:**

- The abbreviation “MHR”, needs to be explained when used for the first time.

**Results:**

- The abbreviation “SHB”, needs to be explained when used for the first time.

**Discussion:**

- “Therefore, this study should be considered…” should be written as: “This study determined the HBV genotype…”
- What do the authors mean by substandard immigration of new strains?
- in vitro should be *in vitro (in italics)*

Is the work clearly and accurately presented and does it cite the current literature?

Yes

If applicable, is the statistical analysis and its interpretation appropriate?

I cannot comment. A qualified statistician is required.

Are all the source data underlying the results available to ensure full reproducibility?

Yes

Is the study design appropriate and is the work technically sound?

Yes

Are the conclusions drawn adequately supported by the results?

Yes

Are sufficient details of methods and analysis provided to allow replication by others?

Partly

Reviewer Expertise:

virology, diagnostics, molecular biology

I confirm that I have read this submission and believe that I have an appropriate level of expertise to confirm that it is of an acceptable scientific standard, however I have significant reservations, as outlined above.

# Reviewer report: Reviewer response for version 1

**DOI:** 10.5256/f1000research.145424.r224163

**Published:** 2023-11-22

**Authors:** Anna McNaughton

**Affiliations:** 1 Nuffield Department of Medicine, University of Oxford Medawar Building, Oxford, England, UK

In this study, the authors sequenced the HBsAg region from a number of samples from individuals co-infected with HIV and HBV. Whilst this data can be informative, there are a number of issues with the analysis, results are not consistent with their metadata and the conclusions need re-evaluating before this manuscript can be reconsidered. The manuscript is also too long in several parts, and should be more focused on relevant information.

 Abstract - Sampling site should be mentioned in the abstract – important to understand how/where these patients were identified, and has a link to the genotype distribution.

 Introduction - Very lengthy, with a large amount of information on HBV biology that is not relevant to this study. The authors should make this section more concise, and focused on HBV/HIV coinfection in the region these patients were sampled from, and the implications of treatment.

 Sampling approach – it is unexpected that 100% of n=50 people tested for HIV would randomly also test positive for HBV via convenience sampling. Were samples screened in advance as part of the clinical testing? The available metadata online (Figshare) also seems to state that 43/50 samples were HBV HBsAg positive, with 7 samples either negative or missing so the authors need to clarify this result. This sampling approach also means that discussions about prevalence and comparing with other studies (in results/particularly in discussion) are not appropriate and should be removed from the study.

 Methods are too detailed and could be more concise.

 Figure 1 is not required in the paper, particularly if sequences were generated.

 PCR Amplification – the authors state that 41/50 amplicons were obtained but that they were not obtained for 11/50 samples – this is 52/50?

 Sequence analyses of overlapping surface/polymerase gene region - The authors state that ‘Phylogenetic tree analysis identified nucleotide sequences from this study as genotype A as depicted in (Figure 2) but the current analysis has issues and cannot be used to inform genotyping assumptions as a result (see below).

 Phylogenetic analysis/Figure 2 - Authors should say what type of phylogenetic analysis this was, and how many study sequences were included in it. What length were these sequences?

 List of reference sequences listed in Figshare does not tally with the sequences in the tree where there appears to be more sequences? Unclear why this is.

 Methods says bootstrap analysis was done but the data is not presented on the tree anywhere.

 There appears to be a few issues with the clustering of the reference sequences in the tree – the clade at the bottom shows genotypes D/B/E/C clustering, but then other genotype B/C sequences can be seen to cluster with genotypes F/H at the top of the tree? Sequences of the same genotype should cluster together and they do not appear to be in this analysis. This analysis needs revising.

 For the mutation analysis, it is unclear what reference sequence the authors were comparing their sequences to? I am not keen of the approach of simply comparing sequences to a reference sequence to determine ‘mutations’ as these are likely to mostly just be reflective of expected sequence diversity. I think looking at a more focused list of sequences with known phenotypic associations would be more informative (as has been done with the resistance-associated mutations). Some rephrasing might help also – for example, saying 47% of sequences had mutations could imply that 53% of sequences were identical in the region? Be clear that these are amino acid differences.

 Table 3 - add the specific drug the mutation provides resistance against.

 Table 4 needs revising to say n= and % for the age groups. Typo of Fisher’s exact test also.

 I think figure 3 can also be removed – this is just showing that sequences more distant from a reference sequence in surface, and also more distant in RT. This is not surprising as the two regions overlap. Are the numbers listed amino acid positions in the sequences – it would be useful to know where in the genome this is?

 The conclusions need re-evaluating after addressing the concerns elsewhere in the paper.

Is the work clearly and accurately presented and does it cite the current literature?

Partly

If applicable, is the statistical analysis and its interpretation appropriate?

Partly

Are all the source data underlying the results available to ensure full reproducibility?

Partly

Is the study design appropriate and is the work technically sound?

No

Are the conclusions drawn adequately supported by the results?

No

Are sufficient details of methods and analysis provided to allow replication by others?

Yes

Reviewer Expertise:

Virology, genomics, epidemiology, viral hepatitis

I confirm that I have read this submission and believe that I have an appropriate level of expertise to state that I do not consider it to be of an acceptable scientific standard, for reasons outlined above.

# Response

**Published:** 2024-04-14

**Authors:** Lorato Mosetsanagape Modise

**Affiliations:** Virology, Sefako Makgatho health Sciences University, Pretoria, Gauteng, South Africa

**Dear Reviewer**

 We appreciate the efforts, comments, and concerns in making the manuscript better. We have gone through the comments and edited the manuscript accordingly in the revised manuscript. The inputs made in response to your comments are listed below.

 In this study, the authors sequenced the HBsAg region from a number of samples from individuals co-infected with HIV and HBV. Whilst this data can be informative, there are several issues with the analysis, results are not consistent with their metadata and the conclusions need re-evaluating.

 before this manuscript can be reconsidered. The manuscript is also too long in several

 parts, and should be more focused on relevant information.

**Reviewer’s comments**:

 Abstract - Sampling site should be mentioned in the abstract – important to understand how/where these patients were identified and has a link to the genotype distribution.

**Response:** *Yes, We have added information in the abstract on where the samples were collected* *(Inkosi Albert Luthuli Central Hospital) and how they were collected (* *Convenience sampling).*

 Introduction - Very lengthy, with a large amount of information on HBV biology that is not relevant to this study. The authors should make this section more concise and focused on HBV/HIV coinfection in the region these patients were sampled from, and the implications of treatment.

***Response*** *: The introduction has been revised and shortened by removing information on the HBV biology and history. We have included information on the* prevalence of *HBV/HIV coinfection in the study region and implication of treatment.*

 Sampling approach – it is unexpected that 100% of n=50 people tested for HIV would randomly also test positive for HBV via convenience sampling. Were samples screened in advance as part of the clinical testing? The available metadata online (Figshare) also seems to state that 43/50 samples were HBV HBsAg positive, with 7 samples either negative or missing so the authors need to clarify this result. This sampling approach also means that discussions about prevalence and comparing with other studies (in results/particularly in discussion) are not appropriate and should be removed from the study.

***Response*** *: The sampling method and sample size is now clarified in the methodology section in page 3 under the title “Study design and population”. Convenience sampling was used to collect 43/50 samples that were previously tested for HIV and HBsAg. The sample size has been revised by reporting the correct size which correspond to the metadata value. The confirmatory screening of HBsAg reported a prevalence of 86% (N=43/50) with 12% (N=6/50) being negative and2% (N=1/50) missing data (Table 1).*

 Reviewer comment: Methods are too detailed and could be more concise.

***Response*** *: The methods have been revised and summarized.*

 Figure 1 is not required in the paper, particularly if sequences were generated.

***Response*** *: Figure has been removed from the paper and included as supplementary data on the repository site.*

 PCR Amplification – the authors state that 41/50 amplicons were obtained but that they were not obtained for 11/50 samples – this is 52/50?

***Response*** *: The result of failed PCR amplification has been corrected to 08/50 instead of 11/50.*

 Sequence analyses of overlapping surface/polymerase gene region

 ‘Phylogenetic tree analysis identified nucleotide sequences from this study as genotype A as depicted in (Figure 2) but the current analysis has issues and cannot be used to inform genotyping assumptions as a result (see below).

 Phylogenetic analysis/Figure 2 - Authors should say what type of phylogenetic analysis this was,

 and how many study sequences were included in it. What length were these sequences?

 List of reference sequences listed in Figshare does not tally with the sequences in the tree?

 there appears to be more sequences. Unclear why this is. Methods says bootstrap analysis was done but the data is not presented on the tree anywhere. There appears to be a few issues with the clustering of the reference sequences in the tree – the clade at the bottom show's genotypes D/B/E/C clustering, but then other genotype B/C sequences can be seen to cluster with genotypes F/H at the top of the tree? Sequences of the same genotype should cluster together, and they do not appear to be in this analysis. This analysis needs revising.

**Response:**

*The prevalence of mutations associated with drug resistance has been revised, to focus only on reporting the mutations associated with specific drug (shown in section titled: Mutations within the polymerase region and table 3)*

*The phylogenetic tree results have been revised. The length these sequences is reported under the PCR amplification results as 547 bp in size and included 24 sequences and 39 reference sequences. The reference sequences listed in the metadata included some of the sequences and not all the reference sequences, but the list has been revised to correspond to the phylogenetic tree reference sequences. The neighbor joining method was used to construct a tree. The clustering of the sequences was revised, and the clustering of the sequences was mixed due to the short PCR product of 547 bp, unlike the full genome which would have given a homogenous clustering pattern. The sequence clustering results have been revised.*

 For the mutation analysis, it is unclear what reference sequence the authors were comparing their

 sequences to? I am not keen of the approach of simply comparing sequences to a reference

 sequence to determine ‘mutations’ as these are likely to mostly just be reflective of expected

 sequence diversity. I think looking at a more focused list of sequences with known phenotypic

 associations would be more informative (as has been done with the resistance-associated

 mutations). Some rephrasing might help also – for example, saying 47% of sequences had

 mutations could imply that 53% of sequences were identical in the region? Be clear that these are amino acid differences.

***Response:*** *The sequences were uploaded into geno2pheno and compared against standard genome with reported mutations using bioinformatics and statistical packages to check for sequence diversity and mutations. We did not investigate the phenotypic associations these mutants have on the virus. The focus was on identifying the mutations in the overlapping pol and S and their clinical implications.*

 Table 3 - add the specific drug the mutation provides resistance against.

***Response*** *: Table 3 has been revised, the initial table has been removed and replaced with the showing specific drug associated with resistance*

 Table 4 needs revising to say n= and % for the age groups. Typo of Fisher’s exact test also. I think figure 3 can also be removed – this is just showing that sequences more distant from a reference sequence in surface, and also more distant in RT. This is not surprising as the two regions overlap. Are the numbers listed amino acid positions in the sequences – it would be useful to know where in the genome this is?

***Response:*** *Table 4 has been removed.*

 The conclusions need re-evaluating after addressing the concerns elsewhere in the paper.

***Response:*** *The conclusion has been revised.*

 The sequences of the studies Q cluster with each other and this is what is shown with my study sequences. There is a clustering between B/C and F/H reference sequences.

 The sequences were compared with the reference sequences on Geno2Pheno, which is an online server tool. Sequence alignment to the deposited HBV consensus sequence of the respective genotype. We looked at genetic mutations, not phenotypic mutations. that will acquire *in vitro *studies which is not the focus of the study.

 Under results section the subheadings 1. mutations within the surface region and 2. mutations within the polymerase region have been combined and reported under the heading: Mutations analysis.

# Reviewer report: Reviewer response for version 1

**DOI:** 10.5256/f1000research.145424.r211197

**Published:** 2023-10-27

**Authors:** Nishi Prabdial-Sing

**Affiliations:** 1 Centre for Vaccines and Immunology, Division of the National Health Laboratory Service, National Institute for Communicable Diseases, Johannesburg, South Africa

The authors undertook a small study to characterize HBV among HIV infected individuals. The evidence and findings that the authors have revealed in the study indicates the necessity to engage in larger studies and population size to understand the extent of HBV sequence variation and diversity seen in South African patients coinfected with HIV. The study provides remarkable technical aspects for many others to undertake studies such as these and the authors are commended on their scientific output.

 There were a few grammatical errors and writing of the manuscript requires improvement. It is imperative that the writing of sequence variation indicates that it is the virus that was sequenced and not host or patient genes, because this can cause confusion to the reader. This has been commented on in the manuscript but the authors need to identify more of these to correct throughout the paper. 

 The discussion is too long and too much information is provided that can be summarized and placed more appropriately in the introduction, especially on the vaccine escape mutations. The discussion should focus on the findings from the study with comparison on other similar studies in other countries or within the same country.

 On the results and discussion, the scatter plot needs to be explained as the X- and Y-axes are not well labeled and the interpretation and relevance of the plot does not seem to be evident in the discussion. The statistical significance is commented on for the RT gene but not shown for the HBsAg gene, but the latter was commented on. The sample size is small to conclude from these statistical significant data. It is recommended that a statistician look at the data and recommend a direction.

 Overall, the manuscript requires major improvement to improve on the aim and discussion and to draw on strengths from the authors' findings.

 The edits and comments are available as a PDF attachment which can be found here.

Is the work clearly and accurately presented and does it cite the current literature?

Yes

If applicable, is the statistical analysis and its interpretation appropriate?

I cannot comment. A qualified statistician is required.

Are all the source data underlying the results available to ensure full reproducibility?

Yes

Is the study design appropriate and is the work technically sound?

Yes

Are the conclusions drawn adequately supported by the results?

Yes

Are sufficient details of methods and analysis provided to allow replication by others?

Partly

Reviewer Expertise:

virology, diagnostics, molecular biology

I confirm that I have read this submission and believe that I have an appropriate level of expertise to confirm that it is of an acceptable scientific standard, however I have significant reservations, as outlined above.

# Response

**Published:** 2024-03-01

**Authors:** Lorato Mosetsanagape Modise

**Affiliations:** Virology, Sefako Makgatho health Sciences University, Pretoria, Gauteng, South Africa

**Dear Reviewer,**

 We appreciate the efforts, comments, and concerns in making the manuscript better. We have gone through the comments and edited the manuscript accordingly in the revised manuscript. The inputs made in response to your comments are listed below.

**Reviewer’s general comments**:

 The authors undertook a small study to characterize HBV among HIV infected individuals. The evidence and findings that the authors have revealed in the study indicates the necessity to engage in larger studies and population size to understand the extent of HBV sequence variation and diversity seen in South African patients coinfected with HIV. The study provides remarkable technical aspects for many others to undertake studies such as these and the authors are commended on their scientific output.

**Reviewer’s comments**: There were a few grammatical errors and writing of the manuscript requires improvement. It is imperative that the writing of sequence variation indicates that it is the virus that was sequenced and not host or patient genes, because this can cause confusion to the reader. This has been commented on in the manuscript, but the authors need to identify more of these to correct throughout the paper.

***Response 1*** *: Yes, we have made the necessary improvement on the grammar and writing of the paper.*

***Response 2: We have made clarification on the use and writing of virus sequence and virus nucleotide sequences and HBV sequence instead of host or patients’ genes.***

**Reviewer’s comments**: The discussion is too long and too much information is provided that can be summarized and placed more appropriately in the introduction, especially on the vaccine escape mutations. The discussion should focus on the findings from the study with comparison on other similar studies in other countries or within the same country.

***Response*** *: Thank you for noting this. We have excluded the opening of the discussion about the seroprevalence. The discussion section has been revised, summarised, and focusing more on the sequence analysis of the viruses (genotyping and mutations analysis).* The information on the *vaccine escape mutations has been moved to the introduction.*

**Reviewer’s specific comments** per section

**Abstract**

**Reviewer’s comments**:

 Add a line on where and how samples were collected?

***Response*** *: The type of samples and the location where the samples were collected is now clarified in the abstract and methodology section in page 3 under the title “Study design and population”*

**Reviewer’s comments**:

 So, here you indicate statistical significance of RT mutations. Were the mutations in the HBsAg statistically significant?  as compared to RT.

***Response*** *: We have provided the statistical significance of association between the RT and HBsAg mutations as outlined in the abstract and results section on page 10 “Association of mutations on the surface and polymerase”*

**Reviewer’s comments**:

 The aim of the study be revised.

***Response:*** We have revised the aim of the study

**Methods**

**Reviewers’ comments**:

 Add a line on where and how samples were collected?

 Response: We added information of how and where the samples were collected. We also describe the sampling method and how the sample size was calculated.

**Results**

**Reviewers’ comments**:

 Is the prevalence of HBsAg correctly reported and how were samples collected?

***Response*** *: The seroprevalence of HBV has been revised by reporting the correct percentage values of HBsAg.*

**Reviewers’ comments**:

 It is not clear what these numbers are? is the first number, the number of mutations found in the age group and the second number is a %? Figure 3 is not well explained as how to read this plot because the X-and Y- axes are not well labelled *.*

***Response*** *: Table 4 and Figure 3 are removed. The probability of association between the RT and SHB which was represented on Table 4 and Figure 3 is reported as depicted on page 10 under section “Association of mutations on the surface and polymerase”* *to report that there was no statistical significance association between the SHB and RT mutations at P > 0.005.*

*Phylogenetic analysis was also revised.*

**Discussion**

**Reviewer’s comments:**

 This is not a valid finding as it may reflect how samples were chosen for the study (previous point above). Also, the sample size is very small and not representative. I would avoid starting the discussion with this. Rather begin at the sequence analyses as this is the strength of the study.

***Response 1*** *: Thank you for noting this. We have excluded the statement on sample size being statistically significant and included it as a study limitation (last paragraph under discussion) on page 11.*

***Response 2*** *: We begin the discussion on the sequence analyses and focus on the genotype identification and diversity.*
